# The influence of biotic and abiotic factors on the bacterial microbiome of gentoo penguins (*Pygoscelis papua*) in their natural environment

**DOI:** 10.1038/s41598-024-66460-9

**Published:** 2024-08-02

**Authors:** Chloe Kaczvinsky, Hila Levy, Stephen Preston, Casey Youngflesh, Gemma Clucas, Heather J. Lynch, Tom Hart, Adrian L. Smith

**Affiliations:** 1 Department of Biology, University of Oxford, 11a Mansfield Road, Oxford, OX1 3SZ UK; 2 Office of Science and Technology Policy, Executive Office of the President, 1650 Pennsylvania Avenue, Washington, DC 20504 USA; 3 UCL School of Pharmacy, University College London, 29-39 Brunswick Square, London, WC1N 1AX UK; 4 Department of Biological Sciences, Clemson University, Clemson, SC 29634 USA; 5 Cornell Lab of Ornithology, 159 Sapsucker Woods Rd., Ithaca, NY 14850 USA; 6 Department of Ecology and Evolution, Stony Brook University, Stony Brook, NY 11794 USA; 7 Institute for Advanced Computational Sciences, Stony Brook University, Stony Brook, NY 11794 USA; 8 Oxford Brookes University, Gypsy Lane, Headington, Oxford OX3 0BP UK

**Keywords:** Pygoscelid penguins, Gentoo penguins, Microbiome, Diet, Monitoring, Ecology, Microbiology, Zoology, Ecology, Ocean sciences

## Abstract

The microbiome is a key factor in the health, well-being, and success of vertebrates, contributing to the adaptive capacity of the host. However, the impact of geographic and biotic factors that may affect the microbiome of wild birds in polar environments is not well defined. To address this, we determined the bacterial 16S rRNA gene sequence profiles in faecal samples from pygoscelid penguin populations in the Scotia Arc, focusing on gentoo penguins. This mesopredatory group breeds in defined colonies across a wide geographic range. Since diet could influence microbiome structure, we extracted dietary profiles from a eukaryotic 18S rRNA gene sequence profile. The bacterial microbiome profiles were considered in the context of a diverse set of environmental and ecological measures. Integrating wide geographic sampling with bacterial 16S and eukaryotic 18S rRNA gene sequencing of over 350 faecal samples identified associations between the microbiome profile and a suite of geographic and ecological factors. Microbiome profiles differed according to host species, colony identity, distance between colonies, and diet. Interestingly there was also a relationship between the proportion of host DNA (in relation to total 18S rRNA gene signal) and the microbiome, which may reflect gut passage time. Colony identity provided the strongest association with differences in microbiome profiles indicating that local factors play a key role in the microbiome structure of these polar seabirds. This may reflect the influence of local transfer of microbes either via faecal-oral routes, during chick feeding or other close contact events. Other factors including diet and host species also associate with variation in microbiome profile, and in at least some locations, the microbiome composition varies considerably between individuals. Given the variation in penguin microbiomes associated with diverse factors there is potential for disruption of microbiome associations at a local scale that could influence host health, productivity, and immunological competence. The microbiome represents a sensitive indicator of changing conditions, and the implications of any changes need to be considered in the wider context of environmental change and other stressors.

## Introduction

The bacterial microbiomes of vertebrates are well established as key components of a healthy host and their composition can be affected by a wide range of factors^1–4^. Experimental approaches with model species have been key to determining the role of microbiome communities on the health^5^, metabolism^6^, and immune response^7^ of animals. While these and many other studies demonstrate the importance of a healthy microbiome in a laboratory environment, the biology of these interactions may be more complicated in wild systems. Studies on humans have associated microbiome changes with a wide range of circumstances, identifying health and nutritional status, disease, host genetics, diet, and geography as key factors that affect microbiome communities^1,3,5,8^. In birds, experimental studies in chickens have demonstrated an important role for diet in determining microbiome composition (e.g.^9,10^), but it is rarely considered in studies of wild vertebrates. Key microbial taxa were detected in sanguivorous finches but were absent in closely related, non-blood-feeding species^11^ and the effect of diet was more important than phylogeny on microbiome composition in Darwin’s finches^4^. Similarly, the microbiome composition of wild-caught great tits was variable and affected by post capture dietary manipulation (seed versus insect) during an 11-day period in captivity^12^, further indicating a role of diet in wild avian microbiomes.

The microbiome of polar species holds particular interest in terms of global biogeographic patterns. Generally, free-living metazoan species distributions follow a pattern of highest diversity in equatorial regions decreasing towards the poles^13^, but this pattern is much less studied in microorganisms^14^, particularly microbiomes that may be more influenced by the host. Wild avian faecal microbiomes are often dominated by four key phyla—Firmicutes, Proteobacteria, Bacteroides, and Actinobacteria^15^. These same phyla also dominate penguin microbiomes^16–19^. A study of Adélie penguins from two parts of the Ross Sea found a significant correlation between genetic distance and the alpha diversity of microbiomes, concluding that these bacteria are influenced by host genetics^16^. Another study comparing the microbiomes of two sympatric Adélie and gentoo penguins found higher concentrations of Actinobacteria and Cyanobacteria in gentoo penguins compared to Adélie penguins^19^. There is also evidence of several known potential pathogens in penguin microbiomes (including *Campylobacter*, *Helicobacter*, and *Streptococcus*) but it is unclear if they are pathogenic in penguins^18^. Importantly, the aforementioned studies were relatively limited in scope, focusing on one or, at most, a few colonies, over relatively restricted distances. Here we investigate a much greater number of penguin microbiomes in multiple colonies across the broad geographic range of the Scotia Arc.

Pygoscelid penguins are a genus of three sympatric species (gentoo [*Pygoscelis papua*], chinstrap [*P. antarcticus*], and Adélie [*P. adeliae*] penguins) with a range from 46° S to 77° S^20–22^. Pygoscelid breeding colonies vary in size from dozens of pairs to half a million or more with varying distances between colonies. Differentiation also exists among these species in their dietary preferences, with Adélie and chinstrap penguins feeding almost exclusively on krill, whereas diets are more diverse in gentoo penguins, including krill, fish, and squid. These variations offer granularity and replication to study the factors that influence penguin microbiomes.

The work presented in this manuscript tests a series of hypotheses on the associations between environmental and biotic factors and microbiome composition in wild gentoo penguins across a large geographic range. We examined a large dataset of > 350 faecal samples (mostly from gentoo penguins but also including samples from chinstrap and Adélie penguins as well as blue-eyed shags [*Phalacrocorax atriceps*], a sympatric species of flying seabird). This study focussed on gentoo penguins with the other pygoscelid penguins and the blue-eyed shags acting as locally derived comparators. Samples were derived from 25 gentoo colonies across the Scotia Arc, covering a substantial geographic and latitudinal ranges. All samples were subject to molecular assessment of microbiome and diet-derived DNA as well as an indicator of the proportion of host DNA in each sample. By combining these measures, we explored the degree to which a range of biotic and abiotic factors are related to variation in the gentoo penguin microbiome.

## Methods

### Sample collection

**Figure 1:**
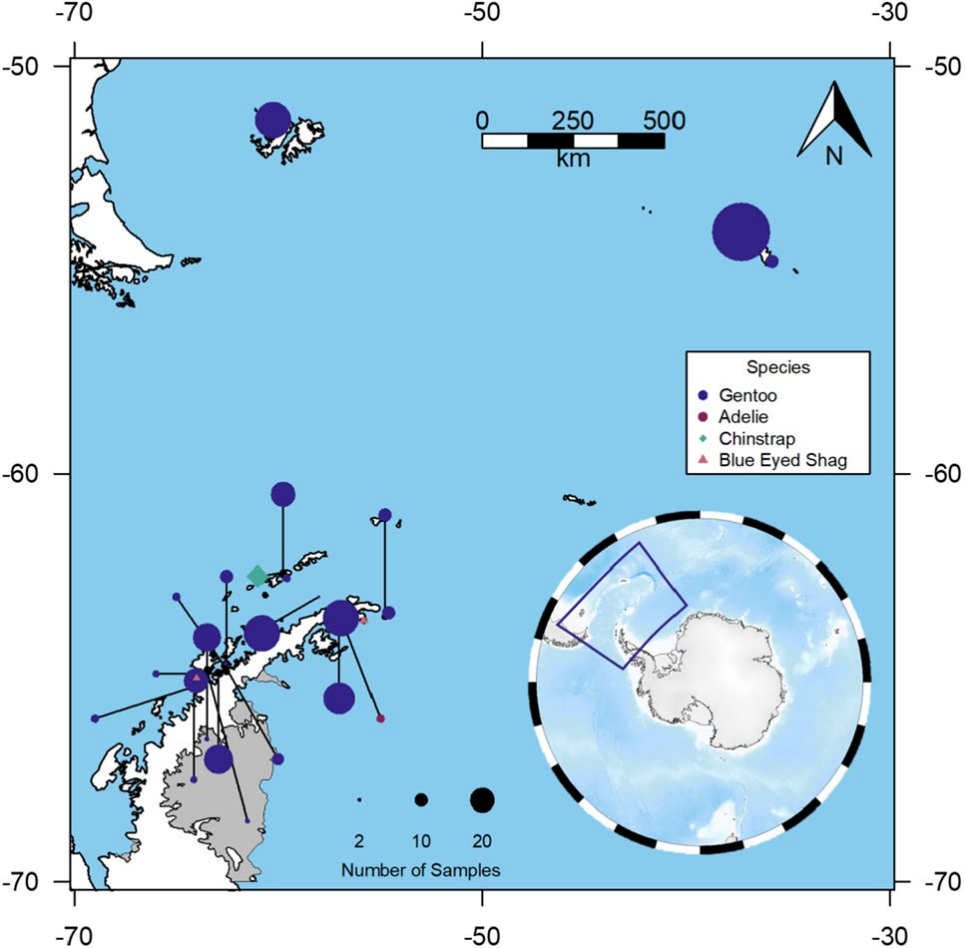
Colony locations and sample size across in the Scotia Arc. Gentoo penguins (n = 337, 23 colonies) are depicted as dark blue circles that vary in diameter according to the number of samples from each location. Adélie penguins (n = 6), chinstrap penguins (n = 20), and blue-eyed shags (n = 18, 3 colonies) are represented by rose square, diamond, and triangle outlines respectively. The insert depicts the location of the Scotia Arc.

Fresh faecal samples (guano) were collected from within 1–2 m of pygoscelid penguin nests distributed throughout the colony to minimize the chances of duplicating samples. Guano samples were discrete and identifiable as a single event each from a single individual (to avoid double sampling of the same individual samples were taken at least 2 nests apart). Samples were collected between the 2015/16 and 2018/19 breeding seasons (November to February) for brush-tailed penguins along the Scotia Arc (Fig. 1 and Supplemental Table 1). Samples were taken from each colony, placed in RNALater® (MFCD03453003)^23^ (at approximately 1:1 volume/volume) in screw-capped Eppendorf® tubes and stored at 4 °C until shipping. These samples were frozen and stored at − 20 °C either in the UK or the Falklands/Malvinas within three weeks. In total, 381 guano samples were collected, with the majority from gentoos (n = 337), and the rest from Adélie or chinstrap penguins (n = 6 and 20 respectively). Eighteen samples were also collected from blue-eyed shags, a sympatric species of flying seabird. Figure 1 summarizes the distribution of sampling across the Scotia Arc.

### DNA extraction and amplicon generation

DNA was extracted using the MoBio PowerSoil® kit (Qiagen, 51804, now branded as Qiagen QIAamp® PowerFecal® DNA Kit). The extraction kit protocol was modified such that the initial lysis step was extended to 12–18 h at 65 °C on a heated shaker block, and the subsequent homogenisation step was carried out on an MP Biomedicals™ FastPrep-24™ (“Marine Sediment” Program: 2 cycles of 40 s at 5.5 m/s with a 5-min pause between cycles). All other steps were according to the manufacturer’s protocol and extracted DNA was stored at − 20 °C.

The V3-V4 region of the prokaryotic 16S rRNA gene was amplified using primers 16S _338F (ACTCCTACGGGAGGCAGCAGT)^24^ and 16S_806R (GGACTACHVGGGTWTCTAAT)^25^. A ~ 170 bp fragment of the V7 region of the eukaryotic nuclear small subunit (SSU) 18S rRNA gene was amplified with the following primers 18S rRNA gene_SSU3_F (GGTCTGTGATGCCCT-TAGATG) and 18S rRNA gene_SSU3_R (GGTGTGTACAAAGGGCAGGG)^26^. In both cases, the forward primer contained a unique 7 nt ‘barcode’ tag to allow pooling of PCR products prior to library preparation. DNA from each guano sample was amplified in triplicate independent PCR reactions with different barcodes to quality control the output. PCR was carried out in 25 µL reactions using the high fidelity Phusion Hot Start Flex DNA polymerase enzyme (New England Biolabs, UK, M0535) according to manufacturer’s recommendations, with 2 µL of 1/10 diluted faecal DNA. Thermal cycling conditions were 98 °C for 1 min; followed by 40 cycles of 98 °C for 15 s, 60 °C for 15 s, 72 °C for 5 s; with a final extension of 72 °C for 30 s. A water control was included for every 48 samples.

PCR products were visualised on a 1% agarose gel stained with SYBR™ Safe DNA Gel Stain. Amplicon pooling was based upon relative band intensity with reference to actual DNA concentration, of a subset of reactions analysed using the Qubit 1X dsDNA High-Sensitivity Assay Kit (Invitrogen, UK, Q33231) using a Qubit Fluorometer. Up to 96 PCR reactions from different samples with individually tagged 16S rRNA gene primers were combined and cleaned using the MinElute PCR purification kit (Qiagen, UK, 28004). Library preparation was performed using the NEBNext® Ultra™ II DNA Library Prep Kit for Illumina® (New England Biolabs, UK, E7103S) according to the manufacturer’s instructions, with no size selection. NEBNext Multiplex Oligos for Illumina (New England Biolabs, UK, E7335S and E7500S) indexing primers were used in order to run multiple libraries on the MiSeq TapeStation (Agilent Genomics) and qPCR (NEBNext® Library Quant Kit for Illumina®, E7630S, New England Biolabs, UK) analyses were used to confirm expected template size and success of the library preparation before loading onto the MiSeq along with 5% phiX at the University of Oxford, Department of Zoology. Sequencing was divided across four MiSeq runs, all using the 600-cycle MiSeq Reagent kit v3 (Illumina, UK), giving 300 nucleotide paired reads that could be merged with sufficient overlap to cover the ~ 468 bp amplicons (with the barcode sequence). Negative controls were included and are available in the data set marked as WC.

### Bioinformatic analysis

Sequence data was processed using a custom python script to de-multiplex sequences, remove primer and barcode sequences. These were then processed on *DADA2*, analysing the data as a pool^27^ to remove chimeras, align sequences, and assign taxonomy using the *DADA2* formatted NCBI database for 16S rRNA gene data and the SILVA 18S rRNA gene database, version 132.99^28^ (script available: 10.6084/m9.figshare.20457378). These assignments were performed^29^ using the reference data sets with Amplicon Sequence Variant (ASV) assignment set to 0.99 identity. Reads were trimmed based on error plots of the pooled data sets to minimize degradation at the end of reads. No uncalled positions (Ns) were allowed and *k*-values for the alignment algorithm were set to 3. After demonstrating that technical triplicates replicated the outputs to a high level, these data were pooled for downstream analyses (see Supplemental Table 2 for metrics). Numbers of reads remaining at each stage of processing are given in Table 1.

**Table 1:** Numbers of reads at each stage of bioinformatic processing.

	Raw	Filtered	Merged	Non-Chimera	Filtering
16S	10,394,014	7,862,089	7,214,834	6,295,746	5,925,116
18S	5,563,002	5,510,560	5,323,332	5,114,051	4,560,848

The number of reads from the input to those remaining after filtering, merging, removing chimeras, and removing those not identified to order respectively. For 18S data, the total number of reads assigned to one of the four food groups equalled 312,447.

We used both Simpson’s (alpha diversity) and Bray–Curtis (beta diversity) measures in our analyses. For alpha diversity, we first tested Simpson, Shannon, and observed richness for the non-rarefied data which exhibited similar patterns (Supplemental Fig. 1). We elected to avoid phylogenetically informed metrics due to the scarcity of data for robust tree generation, (based on a single gene fragment). Bray–Curtis was used as a measure of beta diversity, in part due to the zero-inflated nature of microbiome data, which undermined the use of methods relying on log-transformations^30^. To correct for the influence of read depth data sets were repeatedly (100 replicates) rarefied to 5098 reads/sample for 16S rRNA gene and to 51 reads/sample for 18S rRNA gene (food, 4 components). Model parameters were estimated for each iteration of rarefied data, and the 5th and 95th quantiles, the median estimate, and the median significance value were reported. We selected this method as a balance between the problems of rarefying and normalization. The key problem with rarefying is that it removes informative data^31^. By using repeated rarefaction, we could include rare taxa, without biasing the data with differing read depths and gain a more accurate picture of the variation in and between samples. Although the food data rarefication was performed at 51 reads/sample/iteration, this was sufficient as food comprised only 4 groups. The rarefaction depths were selected to balance retaining reads and samples (See Supplemental Fig. 2A,B).

A generalised linear mixed effects model was developed in the *glmmTMB* package^32^ in R^33^ to test correlation between latitude and alpha diversity for 16S rRNA genes, including colony as a random intercept. This model used a beta distribution to account for the bounds of Simpson’s diversity index between 0 and 1. To examine the connection between ecological metrics and diversity, PERMANOVAs were used to examine these factors against beta diversity, measured by Bray Curtis. Mantel tests were used to test whether geographic distance was an indicator of difference in associated microbiomes. This test used geographic distance estimated using Haversine distance in *geosphere*^34^ and Bray–Curtis dissimilarity matrices as inputs using the mantel function in the *vegan* package in R^35^. Spearman’s distance and 9999 permutations were specified for all tests, except the partial Mantel comparing all three matrices, where the Pearson’s distance was used due to restrictions for input into partial Mantel tests^35,36^. The analyses were performed using the adonis2 and mantel functions respectively in the *vegan* package in R^35^. See Supplemental Fig. 3 for analysis diagram. (Full analysis script: 10.6084/m9.figshare.20457378).

### Ethics approval

All sampling in the Antarctic Treaty Area was carried out in accordance with United Kingdom Home Office guidelines, under permits granted by the United Kingdom Foreign and Commonwealth Office (permits: S3-23/2013, S7 28/2013, S3 04/2014, S7 03/2014, 33/2016, 34/2016, 37/2017), in South Georgia under permits granted by the Government of South Georgia and the South Sandwich Islands (SCI-2014–017, RAP-2015–018, RAP-2016–035 and RAP-2017–034), and in the Falkland Islands/Malvinas under Falkland Islands Government Environmental Planning Department permits (R16.2014, R15 2014, and R04 2017), with ethical approval from the University of Oxford Animal Welfare and Ethical Review Board. Animal handling protocols followed Scientific Committee on Antarctic Research Code of Conduct on the use of Animals for Scientific Purposes in Antarctica. All samples were transported in accordance with applicable export permits and United Kingdom Department for Environment, Food and Rural Affairs import permits.

## Results

**Figure 2:**
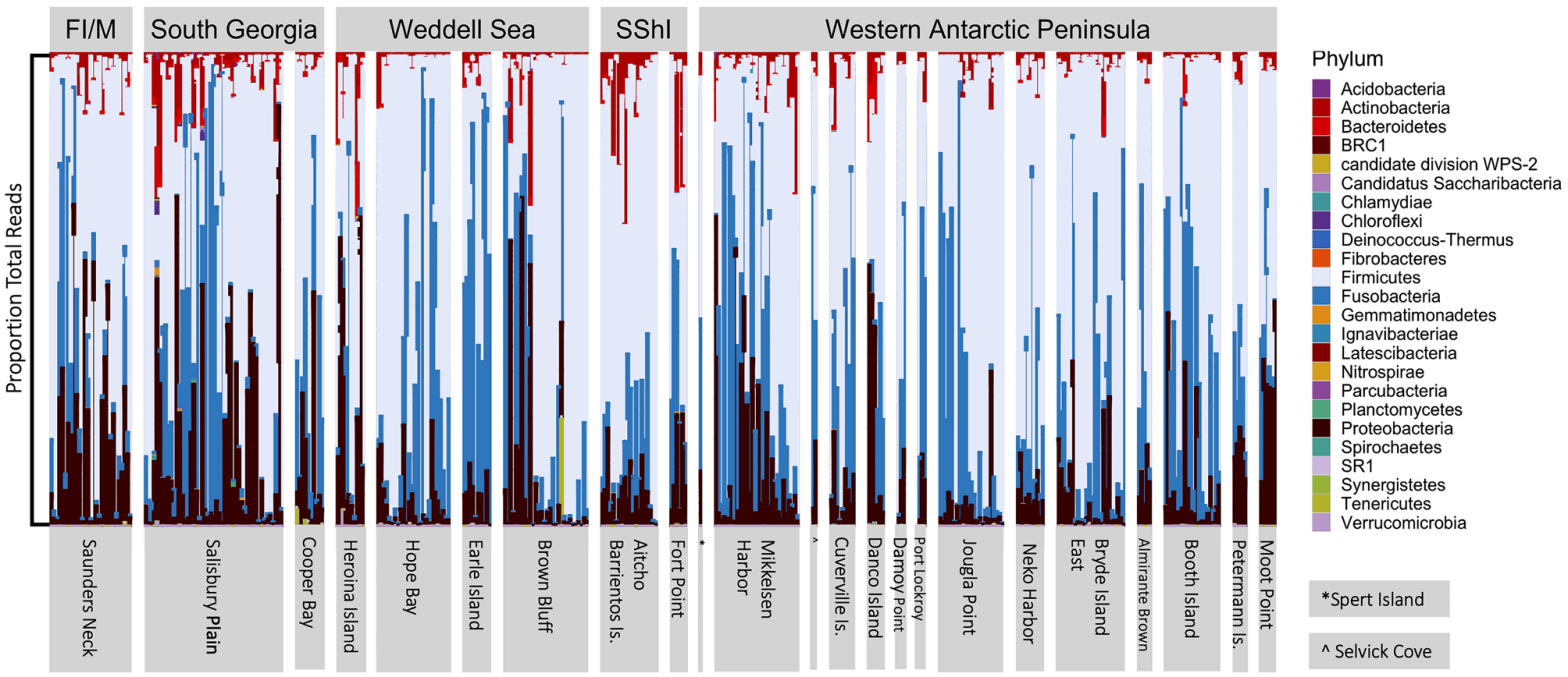
A stacked bar plot depicting the percentage composition of the unrarefied 16S rRNA gene profiles of gentoo penguin samples around the Scotia Sea. Individual microbiomes are reported to Phylum level and samples are arranged into regions (Falkland Islands/Malvinas = FI/M, South Georgia and South Shetland Islands = SShI, Weddell Sea, and Western Antarctic Peninsula). Within these regional groupings, samples are grouped by colony and ordered by latitude, presenting these samples in increasing latitude from the left to the right of the figure. Each bar represents a faecal sample and white space separates colonies.

Examination of 6,009,327 16S rRNA gene sequences identified 8648 ASVs, and 6598 specific bacterial phyla that were retained in the analytical pipeline. The majority of these ASVs (4998) could only be assigned at the level of order and the most abundant were the Clostridiales, Bacilliales, and Pseudomonadales. The remaining 2779 ASVs were identified to one of 188 families. The average (mean) 16S rRNA gene profile of gentoo penguins was dominated by Firmicutes (62%), with significant contributions of Fusobacteria (21%), Proteobacteria (13%), and Actinobacteria (3%) (Fig. 2). All other ASVs represented less than 1% of all reads identifiable to phylum. However, it is important to indicate that the composition of the gentoo faecal 16S rRNA gene profiles was variable and influenced by multiple factors (Supplemental Figs. 4–6) across the 347 samples analysed (See Supplemental Table 1 for all samples). This variation was evident both within and between different colonies (Fig. 2) which provided a basis for considering the factors that may affect microbiome composition across different conditions. (See Supplemental Fig. 7 for an NMDS visualization of the 16S data.)

### Latitude does not correlate with Simpson’s diversity

To test if diversity changed with increased latitude, Simpson’s diversity measures were regressed against year (season) using a beta regression in one model and against latitude and species (gentoo, Adélie, or chinstrap penguin, or blue-eyed shag) in a second model restricted to 2018 data. In both models, colony was included as a random intercept. There was no significant association between Simpson’s diversity and latitude or species in this model (Tables 2, 3).

**Table 2:** Linear mixed effects model examining season and alpha diversity.

	Median	5th percentile	95th percentile	Median * p* value
Intercept	0.74	0.73	0.74	1.0e−09
2016 Season	− 0.042	− 0.051	− 0.034	0.85
2018 Season	0.0061	0.0023	0.010	0.96

Sampling without replacement of the samples to 5098 was repeated 100 times and the model results saved for each run. The median, 5th percentile, and 95th percentile of the estimates are reported as well as the median *p* value. Samples from 2015 were not included in this model as they were limited to a single site. The intercept year was 2015.

**Table 3:** Linear mixed effects model examining alpha diversity with latitude and species.

	Median	5th percentile	95th percentile	Median * p* value
Intercept	0.21	0.17	0.23	0.90
Latitude	− 0.015	− 0.016	− 0.015	0.56
Blue-Eyed Shag	0.64	0.62	0.66	0.20
Chinstrap	− 0.35	− 0.37	− 0.37	0.43
Gentoo	− 0.27	− 0.27	− 0.26	0.45

Sampling without replacement of the samples to 5098 reads was repeated 100 times and the model results saved for each run. The median, 5th percentile, and 95th percentile of the estimates are reported here, as well as the median *p* value. This model is only focused on samples from 2018 as this was the season with the most expansive sampling among different species.

**Figure 3:**
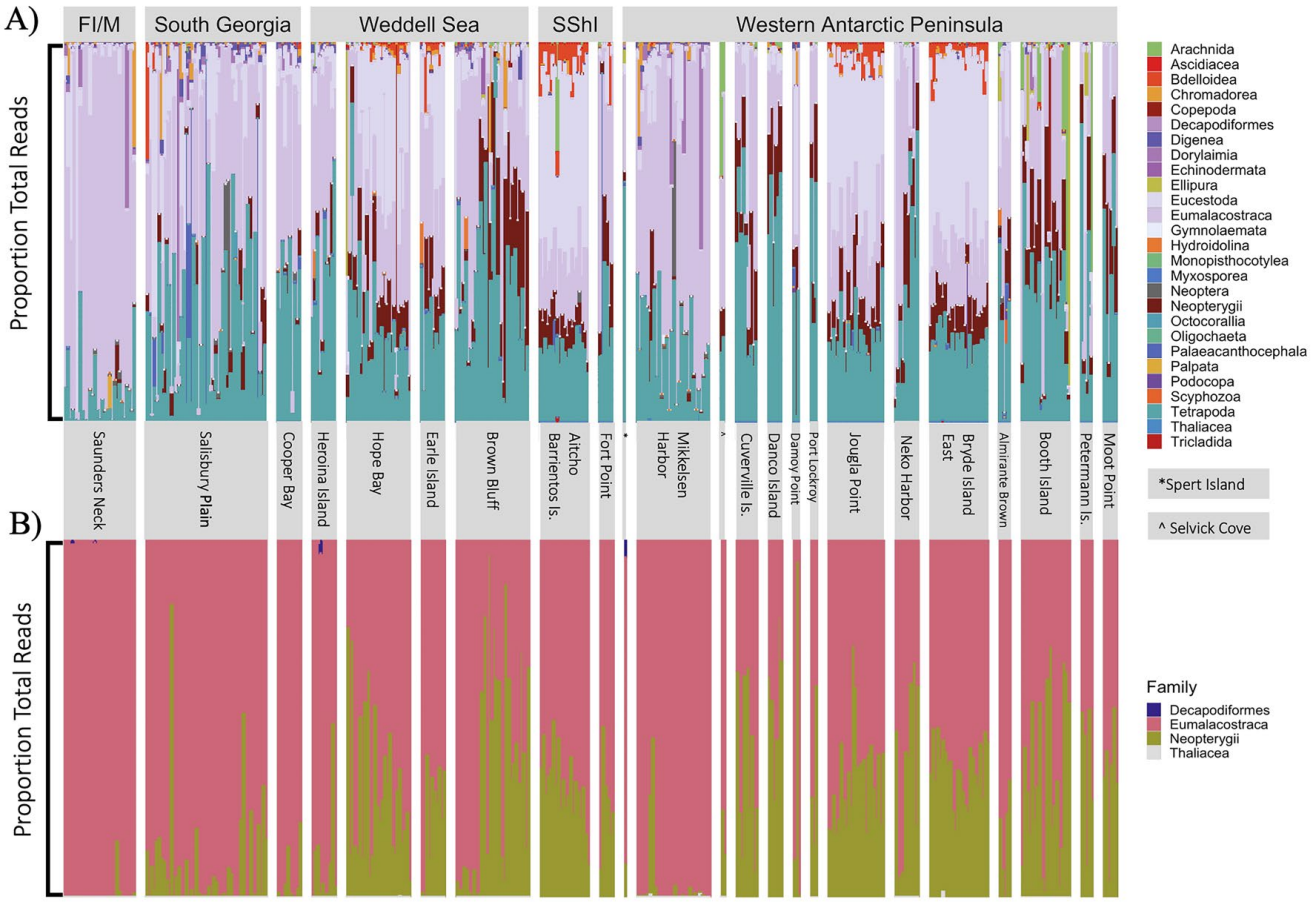
A stacked bar plot depicting the 18S rRNA gene results for gentoo samples, represented as percentages for ease of comparison. Samples are arranged as per this figure by region (top row) and colony (middle row). (**A**) Stacked barplot showing family assignment for all gentoo 18S rRNA gene ASVs in the metazoan data set. (**B**) Stacked barplot showing the proportions of the four determined food groups as ASVs present in faeces of gentoos, where pink represents Eumalacostraca (krill), olive represents Neoptergyii, a subclass of ray-finned fish, dark blue represents Decapodiformes, (squid) and the grey representing Thaliacea (salps). The majority of the estimated diet is attributable to krill, but there is a significant uptake of fish and clear variation between individuals.

**Figure 4:**
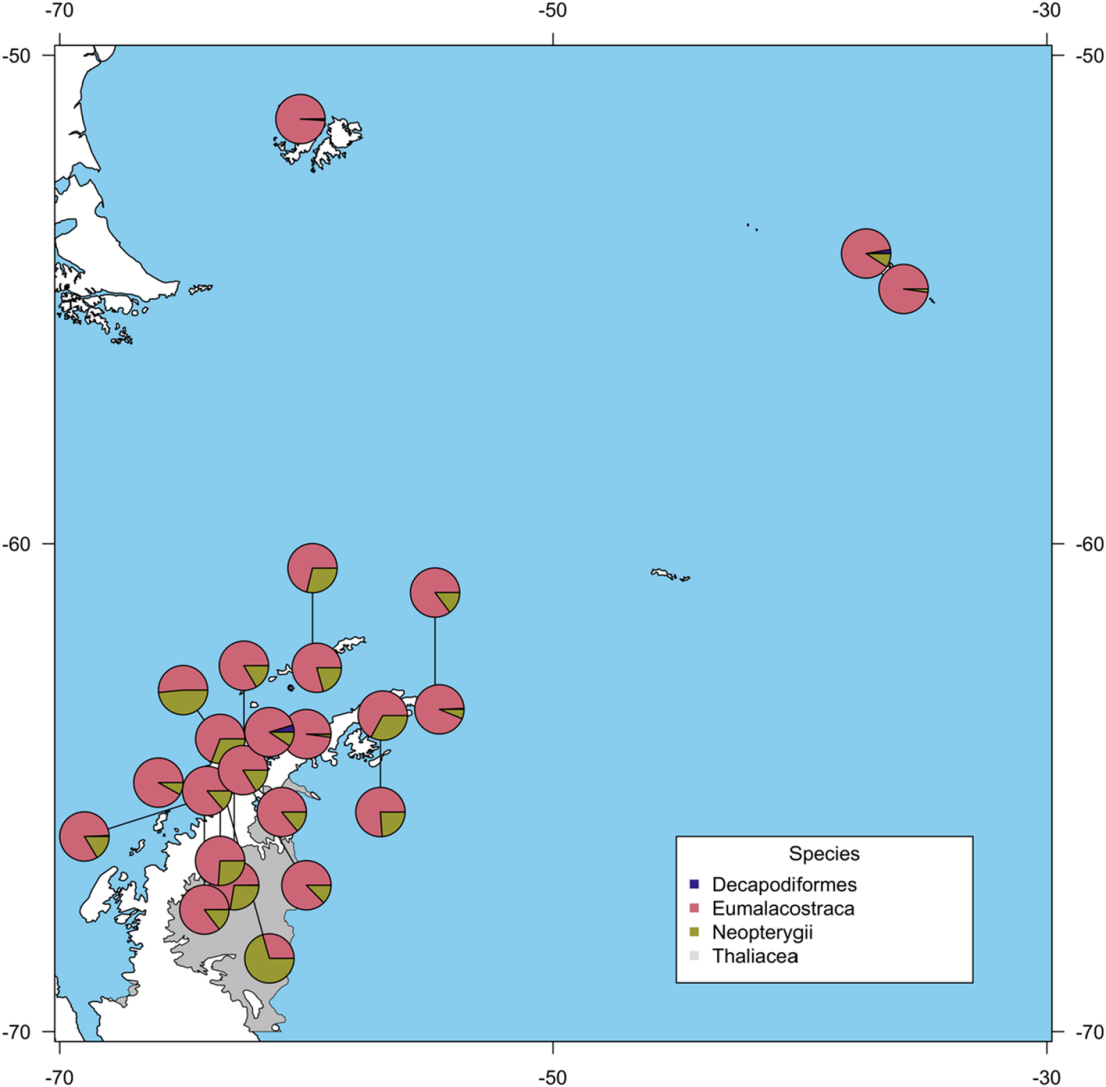
The relative composition of food from gentoo samples across the Scotia Arc as inferred from the 18S rRNA gene data set. Pie charts are constructed as mean proportions of food items in each colony. Pink represents krill (Eumalacostraca) and comprises most of the samples, while olive represents fish (Neopterygii). Where multiple colonies are close together, black lines lead from the expanded colony composition to its location on the map.

One potential determinant in microbiome composition is diet, and since this varies in pygoscelid penguins^20–22^, it was relevant to generate a dietary profile relating to individuals assessed for microbiome composition. A eukaryotic 18S rRNA gene sequencing approach was adopted to identify dietary signatures in the faecal samples. From 5,114,051 total eukaryotic 18S rRNA gene reads, 1759 ASVs were identified as eukaryotic taxa including 255 that could be attributed to metazoans. The faecal 18S rRNA gene signature included a wide variety of organisms (that varied considerably across the sample set) including a large amount of algal (12.7% of raw data) and fungal (5% of raw data) sequences (Fig. 3A). A further 183 ASVs were identified as Opisthokonta, Amoebozoa, or SAR (stramenopiles [heterokonts], alveolates, and Rhizaria), unranked clades below eukaryotes. Many of the non-metazoans were most likely derived from the environment (water) and since diet was the focus of this study, all non-metazoan sequences were excluded from further analysis. Of the metazoans, 111 of 255 taxa were identifiable to Class or superclass. A large but variable proportion of host DNA was present in the metazoan 18S rRNA gene sequences. The proportions of host relative to the metazoan dataset were retained as a potential explanatory variable for other analyses (proportion of host may relate to gut passage time). Finally, the ASVs considered for more detailed analysis were restricted to those identified as food (Eumalacostraca, Decapodiformes, Neopterygii, and Thaliacea), the proportions of which varied across the gentoo faecal 18S rRNA gene dataset and across the Scotia Arc (Figs. 3B, 4). The two most prominent groups in the sample set related to Eumalacostraca (krill) and Neoptergyii (a subclass of ray-finned fish) with a few individuals also containing significant proportions of Decapodiformes (squid) (Fig. 3B). Very small amounts of Thaliacea (salps) were detected in a small number of samples but these have been retained in the dietary group as penguins are known to occasionally consume these as a prey species^37^. (See Supplemental Fig. 7 for an NMDS visualization of the 18S data.)

### Beta diversity variation according to a range of ecological factors

To test the relationship between ecological factors and diversity in microbiome or dietary signatures, Bray–Curtis dissimilarity was used as the dependent variable in the PERMANOVA models against species, colony, percent crustacean, and the percent host (Table 4). Data were also blocked by species and by colony to account for the multiple samples at each colony. This analysis was run twice, first on the whole dataset and then on the gentoo penguins alone. All analyses were performed on rarefied (sub-sampled) data, which were repeated with random sub-sampling 100 times. Median and 90% confidence intervals for the models are reported (Table 4). The results were robust to variations, providing confidence in the models. For the 16S rRNA gene profiles, colony had the strongest correlation with microbiome composition (~ 18%) with species (~ 3%), percent crustacean (~ 1%) and percent host (1.7%) also making significant contributions. Although there were differences between the host species according to this model, we are reluctant to make strong inferences due to the unbalanced dataset (between gentoo and other species). Broadly similar results were obtained when considering gentoos in isolation, with colony being the dominant explanatory factor (18.3%) and percent host (1.9%) remaining significant (Table 4). The percent of crustacean (Eumalacostraca) was not significantly correlated with microbiome when colony was included as a random effect. All other variables were statistically significant, with Pr(> F) values of less than 0.05 (Table 4).

**Table 4:** Reporting the PERMANOVA results of bray Curtis Beta diversity.

Type	Subgroup	Variable	Strata	R^2^ (median)	R^2^ (5th percentile)	R^2^ (95th percentile)	Pr(> F)
16S rRNA Gene Profile	All species	Species		0.036	0.036	0.036	0.001
	All species	Percent Crustacean	Species	0.013	0.013	0.013	0.001
	All species	Colony	Species	0.180	0.180	0.181	0.001
		Percent Crustacean	Species	0.004	0.004	0.004	0.059
	All species	Percent Crustacean	Species	0.014	0.013	0.014	0.001
		Colony	Species	0.172	0.172	0.172	0.001
	All species	Percent Host	Species	0.017	0.017	0.017	0.001
		Colony	Species	0.173	0.172	0.173	0.001
	Gentoos only	Colony		0.183	0.183	0.184	0.001
	Gentoos only	Percent Crustacean	Colony	0.019	0.018	0.019	0.03
	Gentoos only	Percent Host	Colony	0.019	0.019	0.019	0.001
Dietary Profile	All species	Species		0.070	0.064	0.076	0.001
	All species	Colony	Species	0.340	0.326	0.354	0.001
	All species	Percent Host	Species	0.0278	0.024	0.033	0.001
		Colony	Species	0.314	0.301	0.327	0.001
	Gentoos only	Colony		0.396	0.382	0.415	0.001
	Gentoos only	Percent Host		0.053	0.047	0.059	0.001
		Colony		0.346	0.333	0.365	0.001
	Gentoos only	Percent Host	Colony	0.053	0.047	0.059	0.001

The R^2^ was greatest in all models for colony. Dietary profile models only included the four groups identified as part of gentoo diets. PERMANOVA model terms are not interchangeable, so the model examining percent crustacean (Eumalacostrac*a*) and colony have been run twice with the order of terms switched. A dotted border indicates this variable is a continuation from the previous line as part of a multi-variable formula.

The 18S rRNA gene models were similar, ~ 2.8% of the groupings were explained by percent host DNA when included as a factor with colony in the “all species” food model, versus ~ 5% when just examined in gentoos. Colony had the most explanatory power, explaining ~ 30% of the variation in the “all species” food model and ~ 35% in the gentoo model (Table 4). Bird species accounted for ~ 7% of the variation when examined in the “all species” food model. All variables were statistically significant, with Pr(> F) values of less than 0.05 (Table 4). PERMANOVA includes an assessment of intra-site versus inter-site variability. For a visualization with NMDS of both 16S and 18S, please see Supplemental Figs. 7 and 8.

### Beta diversity is correlated with distance

To test for a potential relationship between geographic distance and 16S rRNA gene profiles or dietary profiles (derived from18S rRNA gene sequence profiles), Bray Curtis dissimilarity matrices and distance were compared using Mantel tests. The 16S rRNA gene analysis reported a median Mantel statistic of r = 0.200 and *p* value < 0.0001. For the 18S rRNA gene food dataset, the median Mantel statistic was r = 0.060 and *p* value < 0.0036. Both tests were statistically significant, indicating that samples closer in geographic space were more similar in beta diversity (Table 5).

**Table 5:** Reporting the Mantel results using Bray Curtis Beta diversity.

	16S rRNA genes versus Geography	Sig.*	18S rRNA genes versus Geography	Sig.*	16S rRNA genes versus 18S rRNA genes	Sig.*	Partial	Sig.*
median	0.20	1e−04	0.060	0.0036	− 0.032	0.92	− 0.024	0.84
5th percentile	0.20	1e−04	0.049	0.00069	− 0.038	0.85	− 0.032	0.75
95th percentile	0.20	1e−04	0.072	0.012	− 0.023	0.96	− 0.017	0.91

Both 16S rRNA gene and 18S rRNA gene data sets were correlated with geography, but tests examining correlation between 16S rRNA genes and 18S rRNA genes were non-significant. A partial Mantel test looking for correlation between 16S rRNA genes, 18S rRNA genes, and geographic distance was similarly non-significant. (*Sig. here stands for significance.)

While the models comparing composition of 16S rRNA gene sequence profile with geographic distance and the dietary profile with geographic distance indicate that both diet and microbiome are more similar for samples closer in geographic space, the models do not support a direct correlation between diet and 16S rRNA gene profiles. Similarly, there was no significant correlation between 16S rRNA gene diversity, diet diversity, and geographic distance (Table 5).

## Discussion

The biology of wild Antarctic seabirds provides an opportunity to test the relative influences of biotic and abiotic effects on the microbiome. We show that faecal microbiome profiles were variable among Antarctic seabirds but broadly similar to other wild birds in a review study of over 25 species, including penguins, ostriches, gulls, and geese^38^. In our study, host species explain about 3% of the observed variation in microbiome structure. While other studies have reported on differences in microbiome composition of penguin species in small numbers of individuals at one or a few study sites^17–19,39,40^, our study encompasses over 350 samples and 25 colonies.

Colony identity had the largest effect on both 16S rRNA gene and dietary 18S rRNA gene signatures, though distance was also strongly associated with differences in these profiles. The Adélie penguin faecal microbiome composition has previously been shown to vary between colonies, though there was little evidence for an association with distance between the colonies in the Ross Sea^16^. Our study included a greater diversity of sites across the entire Scotia Arc and focussed on gentoo penguins which exhibit a greater level of philopatry than Adélie penguins^41^. Interestingly, latitude did not have a detectable association with microbiome structure in gentoo penguins. The effect of latitudinal gradients on diversity has been the topic of numerous studies but these have focused on free=living organisms including environmental microbes^42,43^. The internal microbiome of endothermic vertebrates could be considered more likely to be independent of climate (or latitude), but these microbes could still be exposed to latitude-associated environmental pressures during transfer between individuals. The lack of a detectable latitudinal association indicates that the pygoscelid microbiome structure may be more dependent on the host environment than exposure to the external environment. We hypothesise that microbiome transfer between penguins is common (as suggested by the dominant effect of colony identity on microbiome profiles), which might be homogenised by faecal-oral transfer (for example during stone stealing). It is also formally possible that the latitudinal range examined was too small to detect an association.

Our results for the gentoo penguins provided general support for an “isolation by distance” model in both microbiome and diet, and the importance of colony identity on both. Compared with many other factors, colony was one of the largest factors associated with microbiome diversity, accounting for around 18% of the observed variation. While there is support for dietary patterns at the colony or smaller geographic scale, there are no significant biogeographic patterns across the entire range, as indicated by the combined Mantel tests. Individually, both diet and microbiome were more similar among nearby individuals. However, there was no support for co-variation of diet and microbiome. Because the percent crustacean in gentoos was correlated with microbiome within colony, it seems that there is some influence of diet on microbiome, simply not one that appears at the broader geographical scale examined in the Mantel tests. While there is the potential confounding factor of increased relatedness within colonies due to the philopatric lifecycle of gentoo penguins, there is evidence for significant variation in both microbiome profiles and diet within colonies in this and other studies^19,44^. Despite the probability of increased genetic relatedness confounding the results, there is clear variation in diet and microbiome within and between colonies.

In some colonies there was considerable variation in the proportions of krill or bony fish in the diets, yet the colony effect (around 18% explanatory power) remained consistently stronger than the effect of percent crustacean (Eumalacostraca) (statistically significant but only explaining 2% of variance in gentoos) on the microbiome profiles. It could be that the microbiome of prey is part of what determines penguin microbiomes, thus making the geographic origin of diet a factor in penguin microbiomes. In contrast, distance and segregation (but not host genetics or environmental factors) were shown to be key drivers of microbiome structure in Antarctic krill^45^. Interestingly, the krill microbiome composition also diverged when populations were housed in different aquaria under identical physical conditions^45^ showing the importance of isolation between populations in the development of divergent microbiomes. While prey species also contain microbiomes, the penguin faecal signature most closely mapped to other avian faecal microbiome compositions (with very different diets) suggesting any direct effect of prey microbiome-derived DNA was minimal. Given the philopatric nature of gentoo penguins, isolation may be a key driver of the colony-based divergence in microbiome composition^46^. It may be difficult to disentangle the independent effects of factors that covary between colonies and across geographic distances. More extensive sampling of key locations might support greater discrimination of factors in the future. Even with these potential complicating factors, the key observation that distance and colony identity represent strong associations with microbiome composition is an important finding.

Diet has been shown to be a factor in shaping the microbiome in a variety of systems^4,8,12,47–49^. Gentoo penguins are a good example where wild individuals vary in their dietary habits with some being considered individual specialists or individual generalists in terms of the primary prey species being targeted^50^. Moreover, the availability of different diet items is variable across the range of gentoo penguins and may also be subject to seasonal and annual variation which may then modify the microbiome and health of gentoo penguin populations. In this study, most of the dietary 18S rRNA gene signature comprised variable proportions of crustaceans and fish with a few individual samples showing high proportions of squid. This pattern is consistent with the known dietary preference of gentoo penguins, with squid being an occasional opportunistic food source in the region^51^. The interrelationship of diet and microbiome is further demonstrated by the correlation of percent crustacean to beta diversity of the microbiome. It is important to note that the dietary signature calculated from faecal samples most likely represents the most recent meal rather than a longer-term diet of the individual, but it is interesting to note that at some sites most individuals contained similar profiles whereas at other sites the dietary signature was more variable. A more detailed analysis of dietary effect might be possible with greater sampling of sites where diet was identified as a variable within a single colony.

Within the 18S rRNA gene signature, we were not surprised to find that a significant proportion of the sequences were derived from the host although, unexpectedly, the proportion was highly variable. When we considered proportion of host in relation to the observed microbiome structure, this was found to have a relatively small (around 4%) but significant association. We propose that this effect may be related to gut passage time. With longer gut passage times (and a longer time since feeding), we might expect a greater proportion of host to other metazoan (mostly diet) DNA, since we would expect digestive processes to reduce the amount of diet-associated DNA. Gut passage time in humans has been shown to affect the microbiome structure^52^. It could be useful to test the correlation of host DNA percentage with the colour of the faeces in the sample, as green faeces have been attributed to higher amounts of bile and longer fasts^40,53^. Interestingly, the gut microbiomes of a range of penguins were shown to alter during the moult-fast period^39,40^, although with king penguins the changes were more dramatic than other species; a finding proposed to be related to the length of the moult-fast period. It is also important to note that differing passage times might affect detectable dietary 18S rRNA gene signatures with soft-bodied prey being more prone to degradation than those where DNA is protected by hard structures (e.g., bone). There is also evidence of lipid-based bias in diet determination from DNA^54^ that should be taken into account. These factors demonstrate the importance of considering the 18S rRNA gene signatures including host DNA content and how host physiology might affect both diet and microbiome signatures. Given the well-documented differences between gentoo penguins as more generalist predators compared to the more specialist chinstrap and Adélie penguins, a more balanced study of all three species may be useful to segregate the diet/host/colony effects.

## Conclusion

The integration of 16S rRNA gene and dietary 18S rRNA gene sequence data provided a powerful platform to determine the relationship between microbiome and diet and the factors that drive variation in these processes over space and time. In the most studied systems (humans and laboratory animals), the microbiome is well known to be a product of complex interactions including host genetics, location, diet, general health, stress and infection (reviewed in^1,3,48^). This study shows that penguin microbiomes are associated with a similarly complex framework of ecological, physiological, and dietary factors. The most important factors included colony identity and geographic distance between colonies, but there were others (host, species, proportion of host, and dietary composition) that were also associated with microbiome composition. An integrated approach is relevant to our understanding of variation in microbiomes in natural environments. Moreover, these data provide a baseline for continued monitoring of the microbiome in polar birds and may provide a tool to monitor the health of these species as they experience the ongoing effects of environmental change into the future.

### Supplementary Information

Supplementary Information.

## Data Availability

The datasets generated and/or analysed during the current study are available in the Sequence Read Archive (SRA) repository under project PRJNA956456, https://www.ncbi.nlm.nih.gov/bioproject/956456. All scripts used are available at figshare, 10.6084/m9.figshare.20457378.
