# Supplementary material for: The influence of biotic and abiotic factors on the bacterial microbiome of gentoo penguins (Pygoscelis papua) in their natural environment
Source: Sci Rep. 2024 Aug 2;14:17933. doi: 10.1038/s41598-024-66460-9 (PMC11297207; doi:10.1038/s41598-024-66460-9)
Supplement: Supplementary file 1 — Supplementary Information. [file 41598_2024_66460_MOESM1_ESM.docx]

# Supplemental Information

Supplemental Table 1. All samples used in this study.

| **Number of Samples** | **Species** | **Colony** | **Site ID^a^** | **Latitude** | **Longitude** | **Region^b^** | **Season** |
| --- | --- | --- | --- | --- | --- | --- | --- |
| 20 | Chinstrap | Aitcho_Islands | AITC | -62.407 | -59.752 | SSI | 2018 |
| 20 | Gentoo | Aitcho_Islands | AITC | -62.407 | -59.752 | SSI | 2018 |
| 5 | Gentoo | Almirante_Brown | ALMI | -64.896 | -62.870 | WAP | 2018 |
| 20 | Gentoo | Booth_Island | BOOT | -65.067 | -64.026 | WAP | 2018 |
| 10 | Gentoo | Brown_Bluff | BROW | -63.522 | -56.905 | NAP | 2015 |
| 20 | Gentoo | Brown_Bluff | BROW | -63.522 | -56.905 | NAP | 2018 |
| 24 | Gentoo | Bryde_Is_East | BRYE | -64.890 | -62.927 | WAP | 2018 |
| 10 | Gentoo | Cooper_Bay | CBNO | -54.79 | -35.81 | SG | 2015 |
| 9 | Gentoo | Cuverville_Island | CUVE | -64.684 | -62.626 | WAP | 2018 |
| 3 | Gentoo | Damoy_Point | DAMO | -64.8149 | -63.4968 | WAP | 2018 |
| 6 | Gentoo | Danco_Island | DANC | -64.734 | -62.594 | WAP | 2018 |
| 10 | Gentoo | Earl_Island | EARL | -63.483 | -54.783 | NAP | 2016 |
| 6 | Gentoo | Fort_Point | FORT | -62.542 | -59.580 | SSI | 2018 |
| 10 | Gentoo | Heroina_Island | HERO | -63.394 | -54.608 | NAP | 2016 |
| 6 | Adelie | Hope_Bay | HOPE | -63.397 | -56.998 | NAP | 2018 |
| 26 | Gentoo | Hope_Bay | HOPE | -63.397 | -56.998 | NAP | 2018 |
| 5 | Blue_Eyed_Shag | Jougla_Point | JOUG | -64.827 | -63.493 | WAP | 2018 |
| 23 | Gentoo | Jougla_Point | JOUG | -64.827 | -63.493 | WAP | 2018 |
| 3 | Gentoo | Port_Lockroy | LOCK | -64.823 | -63.484 | WAP | 2018 |
| 10 | Gentoo | Mikkelsen_Harbor | MIKK | -63.902 | -60.791 | WAP | 2015 |
| 20 | Gentoo | Mikkelsen_Harbor | MIKK | -63.902 | -60.791 | WAP | 2018 |
| 6 | Gentoo | Moot_Point | MOOT | -65.206 | -64.078 | WAP | 2018 |
| 10 | Gentoo | Neko_Harbor | NEKO | -64.838 | -62.533 | WAP | 2018 |
| 6 | Blue_Eyed_Shag | Paulet_Island | PAUL | -63.580 | -55.788 | NAP | 2018 |
| 5 | Gentoo | Petermann_Island | PETE | -65.176 | -64.137 | WAP | 2018 |
| 13 | Gentoo | Salisbury_Plain | SALI | -54.063 | -37.329 | SG | 2015 |
| 15 | Gentoo | Salisbury_Plain | SALI | -54.063 | -37.329 | SG | 2017 |
| 21 | Gentoo | Salisbury_Plain | SALI | -54.063 | -37.329 | SG | 2018 |
| 2 | Gentoo | Selvick_Cove | SELV | -64.641 | -62.569 | WAP | 2018 |
| 10 | Gentoo | Saunders_Neck | SNEC | -60.241 | -51.307 | FI | 2015 |
| 19 | Gentoo | Saunders_Neck | SNEC | -60.241 | -51.307 | FI | 2018 |
| 1 | Gentoo | Spert_Island | SPER | -63.861 | -60.978 | WAP | 2018 |
| 7 | Blue_Eyed_Shag | Tetrad_Islands | TETR | -63.918 | -60.734 | WAP | 2018 |

**^a^** Site ID is the standardized four-letter code.

**^b^**Region indicates the general area of the site as follows: SSI (South Sandwich Islands), FI (Falkland Islands/Malvinas), SG (South Georgia), NAP (Northern Antarctic Peninsula/Weddell Sea), WAP (Western Antarctic Peninsula).

Supplemental Table 2. Metrics for Triplicate Analysis.

16S Samples

| **Sample^a^** | **Mean** | **Std Dev.** |
| --- | --- | --- |
| APHOPE18s085R | 0.48138268 | 0.02110283 |
| APHOPE18s086R | 0.41574457 | 0.09970095 |
| APHOPE18s087R | 0.50155675 | 0.29167085 |
| APHOPE18s088R | 0.86674656 | 0.07085066 |
| APHOPE18s089R | 0.91060658 | 0.0304523 |
| APHOPE18s095R | 0.74207708 | 0.25566228 |
| BESHJOUG18s575R | 0.8564419 | 0.02729098 |
| BESHJOUG18s576R | 0.82089736 | 0.12949757 |
| BESHJOUG18s577R | 0.74347315 | 0.01930452 |
| BESHJOUG18s578R | 0.78901691 | 0.05418444 |
| BESHJOUG18s579R | 0.79940504 | 0.05753791 |
| BESHPAUL18s096R | 0.77765772 | 0.12383316 |
| BESHPAUL18s097R | 0.90354861 | 0.07295099 |
| BESHPAUL18s098R | 0.93449799 | 0.00071252 |
| BESHPAUL18s099R | 0.93835847 | 0.01288352 |
| BESHPAUL18s100R | 0.93077747 | 0.01569247 |
| BESHPAUL18s101R | 0.89487727 | 0.01254377 |
| CPAITC18s428R | 0.36563712 | 0.15512698 |
| CPAITC18s429R | 0.33136582 | 0.38525393 |
| CPAITC18s430R | 0.61156879 | 0.18057657 |
| CPAITC18s431R | 0.59787759 | 0.24251259 |
| CPAITC18s432R | 0.90743522 | 0.03912449 |
| CPAITC18s433R | 0.88351154 | 0.11063915 |
| CPAITC18s434R | 0.65661268 | 0.15167344 |
| CPAITC18s435R | 0.70645936 | 0.02308536 |
| CPAITC18s436R | 0.69275272 | 0.06044632 |
| CPAITC18s437R | 0.48559308 | 0.34424917 |
| CPAITC18s438R | 0.3469437 | 0.52391504 |
| CPAITC18s439R | 0.9052929 | 0.09019334 |
| CPAITC18s440R | 0.77755423 | 0.10724207 |
| CPAITC18s441R | 0.64201467 | 0.02899399 |
| CPAITC18s442R | 0.74543411 | 0.16710329 |
| CPAITC18s443R | 0.93274454 | 0.02195853 |
| CPAITC18s444R | 0.84030367 | 0.1281665 |
| CPAITC18s445R | 0.56464228 | 0.10012819 |
| CPAITC18s446R | 0.59132824 | 0.30698013 |
| CPAITC18s447R | 0.91105618 | 0.07619754 |
| GPAITC18s448R | 0.79569967 | 0.07812322 |
| GPAITC18s449R | 0.86397335 | 0.06452285 |
| GPAITC18s450R | 0.75714308 | 0.14104569 |
| GPAITC18s451R | 0.54341245 | 0.03017972 |
| GPAITC18s452R | 0.90120729 | 0.02861124 |
| GPAITC18s453R | 0.58922483 | 0.06274595 |
| GPAITC18s454R | 0.79208997 | 0.01192095 |
| GPAITC18s455R | 0.69439785 | 0.25086172 |
| GPAITC18s456R | 0.93001553 | 0.02917117 |
| GPAITC18s457R | 0.77382604 | 0.12829834 |
| GPAITC18s458R | 0.80019834 | 0.05017106 |
| GPAITC18s459R | 0.69246265 | 0.05441826 |
| GPAITC18s460R | 0.88391643 | 0.02305881 |
| GPAITC18s461R | 0.56747123 | 0.14343539 |
| GPAITC18s462R | 0.84240014 | 0.07783859 |
| GPAITC18s463R | 0.74052346 | 0.04945331 |
| GPAITC18s464R | 0.76411415 | 0.12333366 |
| GPAITC18s465R | 0.73533323 | 0.06113158 |
| GPAITC18s466R | 0.41643913 | 0.12705056 |
| GPAITC18s467R | 0.53520361 | 0.01492713 |
| GPALMI18s046R | 0.86040641 | 0.04110618 |
| GPALMI18s047R | 0.83368846 | 0.00199373 |
| GPALMI18s052R | 0.73839644 | 0.02302204 |
| GPALMI18s053R | 0.85291191 | 0.02810455 |
| GPALMI18s054R | 0.88433727 | 0.0037888 |
| GPBR15f001 | 0.78680027 | 0.02018631 |
| GPBR15f002 | 0.5003052 | 0.01234854 |
| GPBR15f003 | 0.88786508 | 0.05467389 |
| GPBR15f004 | 0.39016867 | 0.03469783 |
| GPBR15f005 | 0.74986746 | 0.04837295 |
| GPBR15f006 | 0.66792225 | 0.01354724 |
| GPBR15f007 | 0.84698807 | 0.03044368 |
| GPBR15f008 | 0.76368288 | 0.0073357 |
| GPBR15f009 | 0.63797017 | 0.01770153 |
| GPBR15f010 | 0.86122382 | 0.02210729 |
| GPBROW18s508R | 0.06788269 | 0.00723815 |
| GPBROW18s509R | 0.70623544 | 0.03428846 |
| GPBROW18s510R | 0.47615781 | 0.03133459 |
| GPBROW18s511R | 0.72205824 | 0.01995452 |
| GPBROW18s512R | 0.23854026 | 0.05165886 |
| GPBROW18s513R | 0.35466275 | 0.16753019 |
| GPBROW18s514R | 0.60082296 | 0.02953072 |
| GPBROW18s515R | 0.46138963 | 0.02748902 |
| GPBROW18s516R | 0.16097068 | 0.03243716 |
| GPBROW18s517R | 0.74610938 | 0.00801026 |
| GPBROW18s518R | 0.7167194 | 0.0050425 |
| GPBROW18s519R | 0.2810319 | 0.01252235 |
| GPBROW18s520R | 0.35423751 | 0.03397911 |
| GPBROW18s521R | 0.41620607 | 0.02062777 |
| GPBROW18s522R | 0.03452494 | 0.01389198 |
| GPBROW18s523R | 0.08976463 | 0.01971711 |
| GPBROW18s524R | 0.63618367 | 0.02556051 |
| GPBROW18s525R | 0.31665619 | 0.00921864 |
| GPBROW18s526R | 0.63774323 | 0.02155899 |
| GPBROW18s527R | 0.61237262 | 0.01627355 |
| GPBRYE18s048R | 0.90498089 | 0.0046183 |
| GPBRYE18s049R | 0.80731219 | 0.01525982 |
| GPBRYE18s055R | 0.78200828 | 0.02067487 |
| GPBRYE18s056R | 0.90861358 | 0.0101553 |
| GPBRYE18s057R | 0.9250326 | 0.00108761 |
| GPBRYE18s389R | 0.65857201 | 0.04800479 |
| GPBRYE18s390R | 0.48260134 | 0.02488924 |
| GPBRYE18s391R | 0.44402066 | 0.14832865 |
| GPBRYE18s392R | 0.36562377 | 0.05076476 |
| GPBRYE18s393R | 0.51517806 | 0.00515954 |
| GPBRYE18s394R | 0.55990816 | 0.00713159 |
| GPBRYE18s395R | 0.744151 | 0.09650346 |
| GPBRYE18s396R | 0.50054748 | 0.03274357 |
| GPBRYE18s397R | 0.71735067 | 0.06019582 |
| GPBRYE18s398R | 0.77374967 | 0.00959904 |
| GPBRYE18s399R | 0.65924495 | 0.03180428 |
| GPBRYE18s400R | 0.94127859 | 0.00230368 |
| GPBRYE18s401R | 0.72302201 | 0.01471076 |
| GPBRYE18s402R | 0.88130324 | 0.0090079 |
| GPBRYE18s403R | 0.58348972 | 0.0239039 |
| GPBRYE18s404R | 0.6632736 | 0.01823278 |
| GPBRYE18s405R | 0.67127031 | 0.19969673 |
| GPBRYE18s406R | 0.65187454 | 0.0962776 |
| GPBRYE18s407R | 0.67590688 | 0.03435108 |
| GPCOOP15f001 | 0.62912082 | 0.05436841 |
| GPCOOP15f002 | 0.8097549 | 0.01408295 |
| GPCOOP15f003 | 0.80393305 | 0.01843821 |
| GPCOOP15f004 | 0.72185096 | 0.08098824 |
| GPCOOP15f005 | 0.71089513 | 0.06916416 |
| GPCOOP15f006 | 0.60994064 | 0.07829512 |
| GPCOOP15f007 | 0.83143998 | 0.01932449 |
| GPCOOP15f008 | 0.45851424 | 0.0522181 |
| GPCOOP15f009 | 0.67764473 | 0.00549372 |
| GPCOOP15f010 | 0.57198886 | 0.07766614 |
| GPCUVE18s037R | 0.85979172 | 0.01671723 |
| GPCUVE18s038R | 0.9210019 | 0.00515167 |
| GPCUVE18s039R | 0.69375593 | 0.04410737 |
| GPCUVE18s040R | 0.6626311 | 0.02617245 |
| GPCUVE18s041R | 0.92640676 | 0.01255893 |
| GPCUVE18s042R | 0.89733586 | 0.00376708 |
| GPCUVE18s043R | 0.70739208 | 0.0196903 |
| GPCUVE18s044R | 0.47617539 | 0.04870432 |
| GPCUVE18s045R | 0.82060868 | 0.00720328 |
| GPDAMO18s058R | 0.82930576 | 0.01063361 |
| GPDAMO18s059R | 0.86426388 | 0.09544888 |
| GPDAMO18s060R | 0.73525826 | 0.07344669 |
| GPDANC18s001R | 0.92048227 | 0.00195845 |
| GPDANC18s002R | 0.93919016 | 0.00519947 |
| GPDANC18s003R | 0.90320458 | 0.00352829 |
| GPDANC18s004R | 0.74575599 | 0.0252434 |
| GPDANC18s005R | 0.83936947 | 0.01387301 |
| GPDANC18s006R | 0.62064996 | 0.03132324 |
| GPEARL16f001R | 0.80318354 | 0.02020422 |
| GPEARL16f002R | 0.67923174 | 0.01238194 |
| GPEARL16f003R | 0.82922507 | 0.01245429 |
| GPEARL16f004R | 0.77252607 | 0.00634271 |
| GPEARL16f005R | 0.45393855 | 0.09966901 |
| GPEARL16f006R | 0.88483069 | 0.0052538 |
| GPEARL16f007R | 0.67457241 | 0.04968445 |
| GPEARL16f008R | 0.59269611 | 0.01090935 |
| GPEARL16f009R | 0.43271106 | 0.04543431 |
| GPEARL16f010R | 0.45174192 | 0.11776156 |
| GPFORT18s028R | 0.50336373 | 0.11650023 |
| GPFORT18s029R | 0.83516346 | 0.01356536 |
| GPFORT18s030R | 0.94487439 | 0.00540113 |
| GPFORT18s034R | 0.62369691 | 0.03251615 |
| GPFORT18s035R | 0.95007319 | 0.00490597 |
| GPFORT18s036R | 0.90619523 | 0.01293106 |
| GPHER16f001R | 0.90738218 | 0.00502442 |
| GPHER16f002R | 0.91861933 | 0.00455387 |
| GPHER16f003R | 0.93091006 | NA |
| GPHER16f004R | 0.77213252 | 0.05219262 |
| GPHER16f005R | 0.46715479 | 0.01963267 |
| GPHER16f006R | 0.5246841 | 0.0172406 |
| GPHER16f007R | 0.13841382 | 0.03286673 |
| GPHER16f008R | 0.90469935 | 0.01034978 |
| GPHER16f009R | 0.76839309 | 0.04976959 |
| GPHER16f010R | 0.44754118 | 0.02777886 |
| GPHOPE18s066R | 0.8963717 | 0.02662478 |
| GPHOPE18s090R | 0.89299454 | 0.01464956 |
| GPHOPE18s091R | 0.79256424 | 0.01524383 |
| GPHOPE18s092R | 0.60525578 | 0.08394587 |
| GPHOPE18s093R | 0.22160736 | 0.05353173 |
| GPHOPE18s094R | 0.39948159 | 0.14147966 |
| GPHOPE18s468R | 0.57727874 | 0.01238172 |
| GPHOPE18s469R | 0.45878615 | 0.01763002 |
| GPHOPE18s470R | 0.71998078 | 0.03068569 |
| GPHOPE18s471R | 0.8136244 | 0.00137973 |
| GPHOPE18s472R | 0.74989257 | 0.00781382 |
| GPHOPE18s473R | 0.82272532 | 0.04529459 |
| GPHOPE18s474R | 0.86411168 | 0.00699854 |
| GPHOPE18s475R | 0.85520184 | 0.01320499 |
| GPHOPE18s476R | 0.68002843 | 0.00730217 |
| GPHOPE18s477R | 0.0729713 | 0.00990648 |
| GPHOPE18s478R | 0.46232561 | 0.02330732 |
| GPHOPE18s479R | 0.52646838 | 0.0407695 |
| GPHOPE18s480R | 0.41093598 | 0.0891824 |
| GPHOPE18s481R | 0.75320663 | 0.06166757 |
| GPHOPE18s482R | 0.75454249 | 0.021976 |
| GPHOPE18s483R | 0.78539763 | 0.00809668 |
| GPHOPE18s484R | 0.69478082 | 0.05947429 |
| GPHOPE18s485R | 0.52010765 | 0.03560739 |
| GPHOPE18s486R | 0.09081625 | 0.02408647 |
| GPHOPE18s487R | 0.68438071 | 0.02605017 |
| GPJOUG18s007R | 0.72225956 | 0.01322423 |
| GPJOUG18s008R | 0.57732393 | 0.03408041 |
| GPJOUG18s009R | 0.55379015 | 0.02243489 |
| GPJOUG18s555R | 0.69560502 | 0.06787588 |
| GPJOUG18s556R | 0.80585956 | 0.00805521 |
| GPJOUG18s557R | 0.67055957 | 0.01010372 |
| GPJOUG18s558R | 0.21339963 | 0.09928869 |
| GPJOUG18s559R | 0.5400424 | 0.03134813 |
| GPJOUG18s560R | 0.82063358 | 0.0395065 |
| GPJOUG18s561R | 0.77408796 | 0.01998779 |
| GPJOUG18s562R | 0.65203168 | 0.06675467 |
| GPJOUG18s563R | 0.70872779 | 0.03414498 |
| GPJOUG18s564R | 0.57580702 | 0.00658421 |
| GPJOUG18s565R | 0.70312971 | 0.05602825 |
| GPJOUG18s566R | 0.70087782 | 0.03446403 |
| GPJOUG18s567R | 0.33763045 | 0.04829229 |
| GPJOUG18s568R | 0.65482316 | 0.03198427 |
| GPJOUG18s569R | 0.79907544 | 0.0119327 |
| GPJOUG18s570R | 0.86171063 | 0.01196561 |
| GPJOUG18s571R | 0.69954408 | 0.02669665 |
| GPJOUG18s572R | 0.5865637 | 0.01835517 |
| GPJOUG18s573R | 0.77713792 | 0.00336394 |
| GPJOUG18s574R | 0.50077359 | 0.17033643 |
| GPLOCK18s010R | 0.50455466 | 0.04438493 |
| GPLOCK18s011R | 0.84818657 | 0.01101493 |
| GPLOCK18s012R | 0.87155973 | 0.00821306 |
| GPMH15f001 | 0.89996411 | 0.02383095 |
| GPMH15f002 | 0.87106927 | 0.00481653 |
| GPMH15f003 | 0.50885537 | 0.0349147 |
| GPMH15f004 | 0.74550712 | 0.00507541 |
| GPMH15f005 | 0.50449567 | 0.02190535 |
| GPMH15f006 | 0.81876993 | 0.00480597 |
| GPMH15f007 | 0.76882182 | 0.01146866 |
| GPMH15f008 | 0.66525025 | 0.09583339 |
| GPMH15f009 | 0.76195846 | 0.00775936 |
| GPMH15f010 | 0.84445222 | 0.0069504 |
| GPMIKK18s369R | 0.77531658 | 0.03216553 |
| GPMIKK18s370R | 0.86487735 | 0.03884358 |
| GPMIKK18s371R | 0.47961773 | 0.0436394 |
| GPMIKK18s372R | 0.84159377 | 0.01957078 |
| GPMIKK18s373R | 0.60058869 | 0.46447036 |
| GPMIKK18s374R | 0.91994081 | 0.02184017 |
| GPMIKK18s375R | 0.67569907 | 0.06519554 |
| GPMIKK18s376R | 0.76056095 | 0.02931767 |
| GPMIKK18s377R | 0.88037446 | 0.00945153 |
| GPMIKK18s378R | 0.7071129 | 0.17474991 |
| GPMIKK18s379R | 0.88435004 | 0.02133428 |
| GPMIKK18s380R | 0.73832377 | 0.02625951 |
| GPMIKK18s381R | 0.59353133 | 0.05398561 |
| GPMIKK18s382R | 0.58883049 | 0.11511962 |
| GPMIKK18s383R | 0.89359427 | 0.00750427 |
| GPMIKK18s384R | 0.46657983 | 0.08600754 |
| GPMIKK18s385R | 0.92876762 | 0.00288443 |
| GPMIKK18s386R | 0.91464211 | 0.02094934 |
| GPMIKK18s387R | 0.95195248 | 0.00797388 |
| GPMIKK18s388R | 0.244856 | 0.12707749 |
| GPMOOT18s050R | 0.84108375 | 0.00442412 |
| GPMOOT18s051R | 0.87541043 | 0.0111506 |
| GPMOOT18s067R | 0.90500691 | 0.00683278 |
| GPMOOT18s068R | 0.91891553 | 0.00793375 |
| GPMOOT18s069R | 0.92867939 | 0.00527228 |
| GPMOOT18s070R | 0.79691267 | 0.01509217 |
| GPNEKO18s061R | 0.68529415 | 0.02996422 |
| GPNEKO18s062R | 0.91445525 | 0.01420272 |
| GPNEKO18s063R | 0.77306785 | 0.08848669 |
| GPNEKO18s064R | 0.77935396 | 0.00350715 |
| GPNEKO18s065R | 0.87064273 | 0.01506281 |
| GPNEKO18s080R | 0.50035829 | 0.02164065 |
| GPNEKO18s081R | 0.69400564 | 0.03905668 |
| GPNEKO18s082R | 0.89200234 | 0.00656304 |
| GPNEKO18s083R | 0.89636697 | 0.00352825 |
| GPNEKO18s084R | 0.8829144 | 0.01569603 |
| GPPETE18s071R | 0.93021051 | 0.0001326 |
| GPPETE18s072R | 0.92194341 | 0.01234893 |
| GPPETE18s073R | 0.90860349 | 0.02607394 |
| GPPETE18s074R | 0.90419705 | 0.01625162 |
| GPPETE18s075R | 0.49841716 | 0.01403345 |
| GPPTCH18s488R | 0.58460855 | 0.01739911 |
| GPPTCH18s489R | 0.86850409 | 0.00325053 |
| GPPTCH18s490R | 0.77962922 | 0.00749144 |
| GPPTCH18s491R | 0.85996332 | 0.00536195 |
| GPPTCH18s492R | 0.5491658 | 0.02665882 |
| GPPTCH18s493R | 0.64678822 | 0.0211475 |
| GPPTCH18s494R | 0.60147262 | 0.01445302 |
| GPPTCH18s495R | 0.94507185 | 0.00747974 |
| GPPTCH18s496R | 0.79768684 | 0.00237429 |
| GPPTCH18s497R | 0.80968571 | 0.01502255 |
| GPPTCH18s498R | 0.23086623 | 0.04442063 |
| GPPTCH18s499R | 0.83302004 | 0.01916026 |
| GPPTCH18s500R | 0.73679021 | 0.0143159 |
| GPPTCH18s501R | 0.56749028 | 0.01152262 |
| GPPTCH18s502R | 0.35770189 | 0.03212128 |
| GPPTCH18s503R | 0.45125487 | 0.05550428 |
| GPPTCH18s504R | 0.64759927 | 0.03333048 |
| GPPTCH18s505R | 0.75330223 | 0.00642371 |
| GPPTCH18s506R | 0.77667658 | 0.03578116 |
| GPPTCH18s507R | 0.73284534 | 0.01159267 |
| GPSALI15f001 | 0.59488286 | 0.06251097 |
| GPSALI15f002 | 0.41863432 | 0.00182417 |
| GPSALI15f003 | 0.4778043 | 0.02102995 |
| GPSALI15f004 | 0.9058331 | 0.01667296 |
| GPSALI15f005 | 0.97824 | 0.00234685 |
| GPSALI15f006 | 0.92124162 | 0.01739854 |
| GPSALI15f007 | 0.76760067 | 0.01321109 |
| GPSALI15f008 | 0.72713389 | 0.00587703 |
| GPSALI15f009 | 0.60960477 | 0.17209125 |
| GPSALI15f010 | 0.6982262 | 0.03940885 |
| GPSALI15f011 | 0.42646599 | 0.10761076 |
| GPSALI15f012 | 0.87449315 | 0.02041883 |
| GPSALI15f013 | 0.80687829 | 0.0536351 |
| GPSALI17f001 | 0.63029803 | 0.02794331 |
| GPSALI17f002 | 0.37379004 | 0.00612145 |
| GPSALI17f003 | 0.74449755 | 0.02022386 |
| GPSALI17f004 | 0.53719756 | 0.02805102 |
| GPSALI17f005 | 0.83811679 | 0.03941844 |
| GPSALI17f006 | 0.51444406 | 0.1076852 |
| GPSALI17f007 | 0.09520009 | 0.04093985 |
| GPSALI17f008 | 0.91950366 | 0.02206736 |
| GPSALI17f009 | 0.5459838 | 0.03525071 |
| GPSALI17f010 | 0.17615977 | 0.08624928 |
| GPSALI17f011 | 0.19506836 | 0.05541315 |
| GPSALI17f012 | 0.18060645 | 0.0283507 |
| GPSALI17f013 | 0.58099538 | 0.04066706 |
| GPSALI17f014 | 0.60345609 | 0.20153362 |
| GPSALI17f015 | 0.49115986 | 0.00229352 |
| GPSALI18s601R | 0.88928517 | 0.04008047 |
| GPSALI18s602R | 0.81578101 | 0.0593572 |
| GPSALI18s603R | 0.72287789 | 0.088231 |
| GPSALI18s604R | 0.54810767 | 0.07138455 |
| GPSALI18s605R | 0.83269526 | 0.06240305 |
| GPSALI18s606R | 0.76338319 | 0.02648491 |
| GPSALI18s607R | 0.45370417 | 0.05529167 |
| GPSALI18s608R | 0.85836208 | 0.00623714 |
| GPSALI18s609R | 0.76509598 | 0.02597403 |
| GPSALI18s610R | 0.75230927 | 0.03098417 |
| GPSALI18s611R | 0.88853322 | 0.00809487 |
| GPSALI18s612R | 0.74796623 | 0.01518004 |
| GPSALI18s613R | 0.4422284 | 0.02355602 |
| GPSALI18s614R | 0.78685713 | 0.04470148 |
| GPSALI18s615R | 0.12932175 | 0.01985723 |
| GPSALI18s616R | 0.82689552 | 0.01072474 |
| GPSALI18s617R | 0.60430472 | 0.05985242 |
| GPSALI18s618R | 0.11442185 | 0.03303654 |
| GPSALI18s619R | 0.82693822 | 0.03044803 |
| GPSALI18s620R | 0.81825008 | 0.00745413 |
| GPSALI18s621R | 0.63873536 | 0.04184636 |
| GPSAUN15f001 | 0.53672114 | 0.04307591 |
| GPSAUN15f002 | 0.17548197 | 0.04532249 |
| GPSAUN15f003 | 0.6879001 | 0.02857929 |
| GPSAUN15f004 | 0.68481101 | 0.00658952 |
| GPSAUN15f005 | 0.55929947 | 0.0276034 |
| GPSAUN15f006 | 0.26060752 | 0.10043548 |
| GPSAUN15f007 | 0.64670261 | 0.03024825 |
| GPSAUN15f008 | 0.76516433 | 0.01732224 |
| GPSAUN15f009 | 0.55615486 | 0.01358464 |
| GPSAUN15f010 | 0.68028399 | 0.02861385 |
| GPSAUN18f001R | 0.75971884 | 0.03787906 |
| GPSAUN18f002R | 0.66478857 | 0.10558953 |
| GPSAUN18f003R | 0.81200292 | 0.04674802 |
| GPSAUN18f004R | 0.88211903 | 0.02371801 |
| GPSAUN18f005R | 0.87790659 | 0.03829418 |
| GPSAUN18f006R | 0.78604241 | 0.10384003 |
| GPSAUN18f007R | 0.82945001 | 0.05003966 |
| GPSAUN18f008R | 0.84661652 | 0.02298996 |
| GPSAUN18f009R | 0.69347091 | 0.01584909 |
| GPSAUN18f010R | 0.84257232 | 0.01167618 |
| GPSAUN18f011R | 0.74141692 | 0.06405973 |
| GPSAUN18f012R | 0.85170713 | 0.03101047 |
| GPSAUN18f013R | 0.79257737 | 0.02989728 |
| GPSAUN18f014R | 0.25608188 | 0.13538801 |
| GPSAUN18f015R | 0.81467116 | 0.05427893 |
| GPSAUN18f016R | 0.79317391 | 0.02881891 |
| GPSAUN18f017R | 0.88241504 | 0.01045703 |
| GPSAUN18f018R | 0.8738636 | 0.01976297 |
| GPSAUN18f019R | 0.31294145 | 0.20019533 |
| GPSAUN18sw020 | 0.87982047 | 0.0290321 |
| GPSELV18s020R | 0.55713174 | 0.03838825 |
| GPSELV18s021R | 0.87789755 | 0.00465537 |
| GPSPRT18s024R | 0.71370616 | 0.10586386 |
| WC | 0.79752704 | 0.11143905 |

**^a^** Samples names are ordered by the two or three letter species code, the four letter site ID, and the 2 number year identification, and the individual information.

18S Samples

| **Samples^a^** | **Mean** | **Std. Dev.** |
| --- | --- | --- |
| APHOPE18s085R | 0.60047161 | 0.16764692 |
| APHOPE18s086R | 0.80052452 | 0.00160497 |
| APHOPE18s087R | 0.49958372 | 0.02559225 |
| APHOPE18s088R | 0.69326557 | 0.133397 |
| APHOPE18s089R | 0.73617583 | 0.04998504 |
| APHOPE18s095R | 0.66926702 | 0.27213396 |
| BESHJOUG18s575R | 0.12328086 | 0.05935364 |
| BESHJOUG18s576R | 0.85354078 | 0.00929709 |
| BESHJOUG18s577R | 0.64873993 | 0.05497437 |
| BESHJOUG18s578R | 0.52687278 | 0.01759195 |
| BESHJOUG18s579R | 0.69034639 | 0.03009588 |
| BESHPAUL18s096R | 0.08102897 | 0.04644051 |
| BESHPAUL18s097R | 0.77458508 | 0.06086778 |
| BESHPAUL18s098R | 0.4754623 | 0.01214094 |
| BESHPAUL18s099R | 0.6680364 | 0.10028201 |
| BESHPAUL18s100R | 0.77865536 | 0.0431553 |
| BESHPAUL18s101R | 0.71673875 | 0.05590957 |
| BESHTETR18s548R | 0.51087684 | 0.40410408 |
| BESHTETR18s549R | 0.67253715 | 0.12839789 |
| BESHTETR18s550R | 0.78762428 | 0.07637568 |
| BESHTETR18s551R | 0.36988375 | 0.02116532 |
| BESHTETR18s552R | 0.72792173 | 0.18590717 |
| BESHTETR18s553R | 0.84392136 | 0.00946442 |
| BESHTETR18s554R | 0.85626496 | 0.02243947 |
| CPAITC18s428R | 0.86716126 | 0.03148217 |
| CPAITC18s429R | 0.77646166 | 0.02056694 |
| CPAITC18s430R | 0.72467638 | 0.20589901 |
| CPAITC18s431R | 0.61432002 | 0.21127755 |
| CPAITC18s432R | 0.72173968 | 0.05127314 |
| CPAITC18s433R | 0.79821806 | 0.06651062 |
| CPAITC18s434R | 0.75933544 | 0.02933772 |
| CPAITC18s435R | 0.84115206 | 0.08769046 |
| CPAITC18s436R | 0.79269354 | 0.171511 |
| CPAITC18s437R | 0.80008481 | 0.03972776 |
| CPAITC18s438R | 0.74941383 | 0.10375841 |
| CPAITC18s439R | 0.62978719 | 0.2091236 |
| CPAITC18s440R | 0.58072892 | 0.35032439 |
| CPAITC18s441R | 0.73321634 | 0.05791123 |
| CPAITC18s442R | 0.73564555 | 0.05226761 |
| CPAITC18s443R | 0.80288363 | 0.03826108 |
| CPAITC18s444R | 0.81564012 | 0.06857793 |
| CPAITC18s445R | 0.7129972 | 0.19488009 |
| CPAITC18s446R | 0.82232295 | 0.05146667 |
| CPAITC18s447R | 0.82598452 | 0.02213781 |
| GPAITC18s448R | 0.71541134 | 0.0619193 |
| GPAITC18s449R | 0.6729985 | 0.0287099 |
| GPAITC18s450R | 0.67212682 | 0.03154179 |
| GPAITC18s451R | 0.73442874 | 0.08469356 |
| GPAITC18s452R | 0.71567509 | 0.08887729 |
| GPAITC18s453R | 0.61591008 | 0.05511413 |
| GPAITC18s454R | 0.69015219 | 0.01911619 |
| GPAITC18s455R | 0.71848678 | 0.03033026 |
| GPAITC18s456R | 0.70834459 | 0.02718677 |
| GPAITC18s457R | 0.69131572 | 0.02915796 |
| GPAITC18s458R | 0.6931619 | 0.03487129 |
| GPAITC18s459R | 0.68163478 | 0.02722495 |
| GPAITC18s460R | 0.70595854 | 0.05393134 |
| GPAITC18s461R | 0.68596786 | 0.04228298 |
| GPAITC18s462R | 0.67870642 | 0.04093175 |
| GPAITC18s463R | 0.69428859 | 0.04477209 |
| GPAITC18s464R | 0.6713176 | 0.035594 |
| GPAITC18s465R | 0.66801042 | 0.04307432 |
| GPAITC18s466R | 0.67422223 | 0.02372942 |
| GPAITC18s467R | 0.71688083 | 0.05156419 |
| GPALMI18s046R | 0.50848845 | 0.03288807 |
| GPALMI18s047R | 0.64270401 | 0.04920682 |
| GPALMI18s052R | 0.6579803 | 0.00524376 |
| GPALMI18s053R | 0.56273978 | 0.03775527 |
| GPALMI18s054R | 0.59716406 | 0.00891585 |
| GPBOOT16f001dc | 0.69659105 | 0.01951099 |
| GPBOOT16f002dc | 0.77984056 | 0.02728002 |
| GPBOOT16f003dc | 0.77580314 | 0.01843975 |
| GPBOOT16f004dc | 0.76424323 | 0.02918077 |
| GPBOOT16f005dc | 0.75447038 | 0.03423223 |
| GPBOOT16f006dc | 0.75067024 | 0.08283698 |
| GPBOOT16f007dc | 0.79196907 | 0.00551636 |
| GPBOOT16f008dc | 0.86003818 | 0.01162463 |
| GPBOOT16f009dc | 0.81081658 | 0.029272 |
| GPBOOT16f010dc | 0.8478063 | 0.01695748 |
| GPBR15f001 | 0.37337748 | 0.2197614 |
| GPBR15f002 | 0.23606932 | 0.06970355 |
| GPBR15f003 | 0.80910584 | 0.06209368 |
| GPBR15f004 | 0.47565004 | 0.18084637 |
| GPBR15f005 | 0.46731204 | 0.05669645 |
| GPBR15f006 | 0.44145157 | 0.11178004 |
| GPBR15f007 | 0.73101025 | 0.12347821 |
| GPBR15f008 | 0.46778916 | 0.26186093 |
| GPBR15f009 | 0.73702077 | 0.08042044 |
| GPBR15f010 | 0.82622074 | 0.03243314 |
| GPBROW18s508R | 0.47995352 | 0.12353886 |
| GPBROW18s509R | 0.51012318 | 0.12021252 |
| GPBROW18s510R | 0.61805602 | 0.02456496 |
| GPBROW18s511R | 0.46726375 | 0.12483679 |
| GPBROW18s512R | 0.28837206 | 0.3129311 |
| GPBROW18s513R | 0.43619119 | 0.22876553 |
| GPBROW18s514R | 0.64410033 | 0.08756395 |
| GPBROW18s515R | 0.54347983 | 0.09641731 |
| GPBROW18s516R | 0.46811846 | 0.17190538 |
| GPBROW18s517R | 0.6019646 | 0.16965764 |
| GPBROW18s518R | 0.61497212 | 0.02731591 |
| GPBROW18s519R | 0.55528098 | 0.16222509 |
| GPBROW18s520R | 0.51494875 | 0.13649122 |
| GPBROW18s521R | 0.47963643 | 0.12084679 |
| GPBROW18s522R | 0.54079516 | 0.09567033 |
| GPBROW18s523R | 0.46622297 | 0.11583688 |
| GPBROW18s524R | 0.30873556 | 0.25482837 |
| GPBROW18s525R | 0.74032239 | 0.09053366 |
| GPBROW18s526R | 0.68302231 | 0.03333789 |
| GPBROW18s527R | 0.44384588 | 0.13012794 |
| GPBRYE16f001dc | 0.88805924 | 0.02378788 |
| GPBRYE16f002dc | 0.76576071 | 0.02032177 |
| GPBRYE16f003dc | 0.81277869 | 0.02904637 |
| GPBRYE16f004dc | 0.57256804 | 0.01671752 |
| GPBRYE16f005dc | 0.5779681 | 0.02583939 |
| GPBRYE16f006dc | 0.84627208 | 0.03358715 |
| GPBRYE16f007dc | 0.67228542 | 0.02227229 |
| GPBRYE16f008dc | 0.7536485 | 0.02492898 |
| GPBRYE16f009dc | 0.81828937 | 0.01626284 |
| GPBRYE16f010dc | 0.7937078 | 0.03207149 |
| GPBRYE18s048R | 0.68343178 | 0.05419438 |
| GPBRYE18s049R | 0.71822371 | 0.05790171 |
| GPBRYE18s055R | 0.68911395 | 0.02001395 |
| GPBRYE18s056R | 0.65969806 | 0.00578865 |
| GPBRYE18s057R | 0.69976892 | 0.04706256 |
| GPBRYE18s389R | 0.73900393 | 0.04852792 |
| GPBRYE18s390R | 0.68941393 | 0.04467276 |
| GPBRYE18s391R | 0.66587335 | 0.03794697 |
| GPBRYE18s392R | 0.63640672 | 0.03240965 |
| GPBRYE18s393R | 0.69832318 | 0.01703583 |
| GPBRYE18s394R | 0.70300893 | 0.04786788 |
| GPBRYE18s395R | 0.66908283 | 0.00930713 |
| GPBRYE18s396R | 0.71366124 | 0.0272049 |
| GPBRYE18s397R | 0.69973212 | 0.05889459 |
| GPBRYE18s398R | 0.67737897 | 0.03284398 |
| GPBRYE18s399R | 0.66643252 | 0.02997447 |
| GPBRYE18s400R | 0.70247601 | 0.05010815 |
| GPBRYE18s401R | 0.68024784 | 0.00992455 |
| GPBRYE18s402R | 0.68431615 | 0.02370352 |
| GPBRYE18s403R | 0.66382898 | 0.03464454 |
| GPBRYE18s404R | 0.67882959 | 0.04488669 |
| GPBRYE18s405R | 0.70161197 | 0.02768637 |
| GPBRYE18s406R | 0.69624941 | 0.01860395 |
| GPBRYE18s407R | 0.76894511 | 0.0116183 |
| GPCOOP15f001 | 0.42999644 | 0.19901182 |
| GPCOOP15f002 | 0.7236941 | 0.07936105 |
| GPCOOP15f003 | 0.88257152 | 0.01088059 |
| GPCOOP15f004 | 0.42187927 | 0.13206908 |
| GPCOOP15f005 | 0.52092252 | 0.20391429 |
| GPCOOP15f006 | 0.71543927 | 0.1403668 |
| GPCOOP15f007 | 0.75267308 | 0.05478122 |
| GPCOOP15f008 | 0.58521933 | 0.0904917 |
| GPCOOP15f009 | 0.6413244 | 0.08499252 |
| GPCOOP15f010 | 0.43710522 | 0.01030117 |
| GPCUVE16f001dc | 0.81909212 | 0.01900715 |
| GPCUVE16f002dc | 0.82895266 | 0.05049819 |
| GPCUVE16f003dc | 0.83707847 | 0.02180125 |
| GPCUVE16f004dc | 0.89042059 | 0.00537021 |
| GPCUVE16f005dc | 0.79479829 | 0.02604022 |
| GPCUVE16f006dc | 0.77400473 | 0.01542185 |
| GPCUVE16f007dc | 0.86395672 | 0.0164007 |
| GPCUVE16f008dc | 0.79803017 | 0.0331714 |
| GPCUVE16f009dc | 0.61138676 | 0.04013971 |
| GPCUVE16f010dc | 0.78313605 | 0.01277329 |
| GPCUVE18s037R | 0.63489429 | 0.01808832 |
| GPCUVE18s038R | 0.69151564 | 0.07948267 |
| GPCUVE18s039R | 0.61076757 | 0.0162165 |
| GPCUVE18s040R | 0.46836928 | 0.13595236 |
| GPCUVE18s041R | 0.61569445 | 0.20376905 |
| GPCUVE18s042R | 0.5867741 | 0.24537551 |
| GPCUVE18s043R | 0.7177933 | 0.06549583 |
| GPCUVE18s044R | 0.51301379 | 0.11975803 |
| GPCUVE18s045R | 0.54662119 | 0.14236312 |
| GPDAMO18s058R | 0.18659983 | 0.01920006 |
| GPDAMO18s059R | 0.41513491 | 0.01957806 |
| GPDAMO18s060R | 0.28541584 | 0.06959956 |
| GPDANC18s001R | 0.71306994 | 0.04611017 |
| GPDANC18s002R | 0.58614256 | 0.05699764 |
| GPDANC18s003R | 0.78122341 | 0.07190369 |
| GPDANC18s004R | 0.60369465 | 0.04782108 |
| GPDANC18s005R | 0.63628621 | 0.0454112 |
| GPDANC18s006R | 0.49818223 | 0.1269357 |
| GPEARL16f001dc | 0.64496681 | 0.05216957 |
| GPEARL16f001R | 0.51334843 | 0.06497375 |
| GPEARL16f002dc | 0.77655496 | 0.04428945 |
| GPEARL16f002R | 0.66847856 | 0.09498662 |
| GPEARL16f003dc | 0.67801716 | 0.13890944 |
| GPEARL16f003R | 0.75299206 | 0.10448056 |
| GPEARL16f004dc | 0.63449162 | 0.02048453 |
| GPEARL16f004R | 0.58228224 | 0.22291143 |
| GPEARL16f005dc | 0.6411561 | 0.07265923 |
| GPEARL16f005R | 0.6497038 | 0.17709567 |
| GPEARL16f006dc | 0.80513226 | 0.05589031 |
| GPEARL16f006R | 0.69472163 | 0.04211201 |
| GPEARL16f007dc | 0.67991539 | 0.21403255 |
| GPEARL16f007R | 0.5357355 | 0.10178355 |
| GPEARL16f008dc | 0.44298294 | 0.22276011 |
| GPEARL16f008R | 0.623297 | 0.03295214 |
| GPEARL16f009dc | 0.445814 | 0.14460436 |
| GPEARL16f009R | 0.67103078 | 0.01210053 |
| GPEARL16f010dc | 0.73792182 | 0.03892897 |
| GPEARL16f010R | 0.45688916 | 0.13013423 |
| GPFORT18s028R | 0.3731311 | 0.22088114 |
| GPFORT18s029R | 0.41392123 | 0.23286498 |
| GPFORT18s030R | 0.71241146 | 0.07192241 |
| GPFORT18s034R | 0.5705119 | 0.09965927 |
| GPFORT18s035R | 0.628867 | 0.03975176 |
| GPFORT18s036R | 0.37324002 | 0.19212983 |
| GPGEOR16f001dc | 0.73795271 | 0.02630322 |
| GPGEOR16f003dc | 0.81005909 | 0.02807745 |
| GPGEOR16f004dc | 0.80251561 | 0.04085648 |
| GPGEOR16f005dc | 0.81763793 | 0.027382 |
| GPGEOR16f006dc | 0.85959904 | 0.01762607 |
| GPGEOR16f007dc | 0.8046883 | 0.01148959 |
| GPGEOR16f008dc | 0.78905467 | 0.04659328 |
| GPGEOR16f009dc | 0.872447 | 0.0358412 |
| GPGEOR16f010dc | 0.51541565 | 0.01171957 |
| GPHER16f001dc | 0.48172764 | 0.05388012 |
| GPHER16f001R | 0.78756034 | 0.06148311 |
| GPHER16f002dc | 0.80170591 | 0.03097826 |
| GPHER16f002R | 0.75870093 | 0.02384975 |
| GPHER16f003dc | 0.79465806 | 0.00672277 |
| GPHER16f003R | 0.80657036 | 0.05398514 |
| GPHER16f004dc | 0.85547668 | 0.03521438 |
| GPHER16f004R | 0.36532303 | 0.0728277 |
| GPHER16f005dc | 0.50025546 | 0.14287651 |
| GPHER16f005R | 0.42776943 | 0.20215994 |
| GPHER16f006dc | 0.59669094 | 0.16348464 |
| GPHER16f006R | 0.31766906 | 0.09759627 |
| GPHER16f007dc | 0.66644671 | 0.06518628 |
| GPHER16f007R | 0.69729997 | 0.08110864 |
| GPHER16f008dc | 0.69783297 | 0.02939564 |
| GPHER16f008R | 0.81479011 | 0.05920639 |
| GPHER16f009dc | 0.63232153 | 0.00595627 |
| GPHER16f009R | 0.43454332 | 0.12434456 |
| GPHER16f010dc | 0.57056115 | 0.04798148 |
| GPHER16f010R | 0.25527255 | 0.063833 |
| GPHOPE18s066R | 0.5525976 | 0.10576635 |
| GPHOPE18s090R | 0.65287878 | 0.1024479 |
| GPHOPE18s091R | 0.50743069 | 0.1411251 |
| GPHOPE18s092R | 0.40768717 | 0.23781732 |
| GPHOPE18s093R | 0.32000005 | 0.28990437 |
| GPHOPE18s094R | 0.29931374 | 0.30637018 |
| GPHOPE18s468R | 0.4056983 | 0.17326789 |
| GPHOPE18s469R | 0.61101113 | 0.08102157 |
| GPHOPE18s470R | 0.33161075 | 0.21123518 |
| GPHOPE18s471R | 0.33186258 | 0.24730019 |
| GPHOPE18s472R | 0.52318047 | 0.12597193 |
| GPHOPE18s473R | 0.27067279 | 0.13326946 |
| GPHOPE18s474R | 0.69214697 | 0.11997338 |
| GPHOPE18s475R | 0.61732611 | 0.1441457 |
| GPHOPE18s476R | 0.66407807 | 0.10947441 |
| GPHOPE18s477R | 0.63879472 | 0.03156814 |
| GPHOPE18s478R | 0.68285299 | 0.06902008 |
| GPHOPE18s479R | 0.72744477 | 0.05885746 |
| GPHOPE18s480R | 0.73044806 | 0.07098066 |
| GPHOPE18s481R | 0.70481739 | 0.08302106 |
| GPHOPE18s482R | 0.71380013 | 0.08778635 |
| GPHOPE18s483R | 0.64330597 | 0.09826017 |
| GPHOPE18s484R | 0.51226715 | 0.17700264 |
| GPHOPE18s485R | 0.74823232 | 0.0927991 |
| GPHOPE18s486R | 0.66936518 | 0.10818687 |
| GPHOPE18s487R | 0.42953719 | 0.17978405 |
| GPJOUG18s007R | 0.62721462 | 0.03668769 |
| GPJOUG18s008R | 0.60118372 | 0.03476684 |
| GPJOUG18s009R | 0.60807149 | 0.03506836 |
| GPJOUG18s555R | 0.68253498 | 0.03557219 |
| GPJOUG18s556R | 0.55541102 | 0.06809086 |
| GPJOUG18s557R | 0.36161444 | 0.17213216 |
| GPJOUG18s558R | 0.60266292 | 0.05984985 |
| GPJOUG18s559R | 0.69853653 | 0.04807629 |
| GPJOUG18s560R | 0.6303542 | 0.02836938 |
| GPJOUG18s561R | 0.66974936 | 0.11291095 |
| GPJOUG18s562R | 0.6029004 | 0.01445034 |
| GPJOUG18s563R | 0.44210533 | 0.15635475 |
| GPJOUG18s564R | 0.67247621 | 0.04345366 |
| GPJOUG18s565R | 0.69656235 | 0.01047508 |
| GPJOUG18s566R | 0.58267851 | 0.06785152 |
| GPJOUG18s567R | 0.65781084 | 0.03515246 |
| GPJOUG18s568R | 0.67779086 | 0.04007032 |
| GPJOUG18s569R | 0.54190189 | 0.10567264 |
| GPJOUG18s570R | 0.6373603 | 0.05323078 |
| GPJOUG18s571R | 0.59857962 | 0.09060407 |
| GPJOUG18s572R | 0.42362576 | 0.17095582 |
| GPJOUG18s573R | 0.66304174 | 0.0261787 |
| GPJOUG18s574R | 0.34074632 | 0.23255485 |
| GPLOCK18s010R | 0.72713819 | 0.12506454 |
| GPLOCK18s011R | 0.57992277 | 0.07295208 |
| GPLOCK18s012R | 0.54721157 | 0.17973478 |
| GPMH15f001 | 0.81162082 | 0.06687951 |
| GPMH15f002 | 0.64198419 | 0.14551916 |
| GPMH15f003 | 0.47559014 | 0.19416939 |
| GPMH15f004 | 0.51647202 | 0.11829856 |
| GPMH15f005 | 0.61915181 | 0.11312625 |
| GPMH15f006 | 0.30680498 | 0.12275952 |
| GPMH15f007 | 0.2199524 | 0.097158 |
| GPMH15f008 | 0.74810787 | 0.04969584 |
| GPMH15f009 | 0.72299757 | 0.03584556 |
| GPMH15f010 | 0.8149492 | 0.0339378 |
| GPMH16f001dc | 0.85326966 | 0.01225284 |
| GPMH16f002dc | 0.85585572 | 0.00377252 |
| GPMH16f003dc | 0.86017509 | 0.00906086 |
| GPMH16f004dc | 0.62869427 | 0.03609568 |
| GPMH16f005dc | 0.86013195 | 0.02598698 |
| GPMH16f006dc | 0.76415354 | 0.03871408 |
| GPMH16f007dc | 0.7414345 | 0.01478122 |
| GPMH16f008dc | 0.52873193 | 0.09792434 |
| GPMH16f009dc | 0.66149029 | 0.01415366 |
| GPMH16f010dc | 0.64522853 | 0.06610295 |
| GPMIKK18s369R | 0.79895919 | 0.00291857 |
| GPMIKK18s370R | 0.80563849 | 0.06131464 |
| GPMIKK18s371R | 0.75809137 | 0.07281109 |
| GPMIKK18s372R | 0.64000549 | 0.01923349 |
| GPMIKK18s373R | 0.81418851 | 0.02167797 |
| GPMIKK18s374R | 0.80381737 | 0.0447908 |
| GPMIKK18s375R | 0.81408418 | 0.05966309 |
| GPMIKK18s376R | 0.79814086 | 0.04845961 |
| GPMIKK18s377R | 0.80302435 | 0.02140709 |
| GPMIKK18s378R | 0.86079678 | 0.02187231 |
| GPMIKK18s379R | 0.80551382 | 0.02917491 |
| GPMIKK18s380R | 0.81125159 | 0.00322236 |
| GPMIKK18s381R | 0.74950825 | 0.06265927 |
| GPMIKK18s382R | 0.79124089 | 0.02538042 |
| GPMIKK18s383R | 0.78192098 | 0.01688794 |
| GPMIKK18s384R | 0.7074762 | 0.04755154 |
| GPMIKK18s385R | 0.79924687 | 0.03126533 |
| GPMIKK18s386R | 0.77887108 | 0.06639264 |
| GPMIKK18s387R | 0.79375167 | 0.01483906 |
| GPMIKK18s388R | 0.80266919 | 0.02959544 |
| GPMOOT16f001dc | 0.70878758 | 0.02378262 |
| GPMOOT16f002dc | 0.72334423 | 0.02605752 |
| GPMOOT16f003dc | 0.76001317 | 0.01342755 |
| GPMOOT16f004dc | 0.76102821 | 0.02842808 |
| GPMOOT16f006dc | 0.81031513 | 0.02696123 |
| GPMOOT16f007dc | 0.73833788 | 0.04753731 |
| GPMOOT16f008dc | 0.76951467 | 0.04887831 |
| GPMOOT16f009dc | 0.59298532 | 0.02237925 |
| GPMOOT16f010dc | 0.73335637 | 0.02017722 |
| GPMOOT18s050R | 0.64610504 | 0.081959 |
| GPMOOT18s051R | 0.7592227 | 0.12105395 |
| GPMOOT18s067R | 0.74465075 | 0.11231753 |
| GPMOOT18s068R | 0.65589879 | 0.04895728 |
| GPMOOT18s069R | 0.67797913 | 0.1203341 |
| GPMOOT18s070R | 0.80329506 | 0.13410317 |
| GPNEKO16f001dc | 0.77378125 | 0.08881424 |
| GPNEKO16f002dc | 0.53146532 | 0.05814267 |
| GPNEKO16f003dc | 0.81034764 | 0.02208866 |
| GPNEKO16f004dc | 0.81869319 | 0.01564473 |
| GPNEKO16f005dc | 0.79154531 | 0.02024082 |
| GPNEKO16f006dc | 0.8018225 | 0.04585793 |
| GPNEKO16f007dc | 0.65566139 | 0.04442302 |
| GPNEKO16f008dc | 0.8353471 | 0.02349613 |
| GPNEKO16f009dc | 0.82443042 | 0.04180539 |
| GPNEKO16f010dc | 0.67009959 | 0.01436114 |
| GPNEKO18s061R | 0.65310871 | 0.02765017 |
| GPNEKO18s062R | 0.6544181 | 0.0226092 |
| GPNEKO18s063R | 0.69628834 | 0.0932666 |
| GPNEKO18s064R | 0.70000624 | 0.07827793 |
| GPNEKO18s065R | 0.57551075 | 0.13919317 |
| GPNEKO18s080R | 0.59058547 | 0.03540924 |
| GPNEKO18s081R | 0.72194096 | 0.08681431 |
| GPNEKO18s082R | 0.7417642 | 0.07631518 |
| GPNEKO18s083R | 0.57199466 | 0.06796386 |
| GPNEKO18s084R | 0.43576007 | 0.20880427 |
| GPPETE16f001dc | 0.57782395 | 0.15327734 |
| GPPETE16f002dc | 0.68520707 | 0.01102204 |
| GPPETE16f003dc | 0.86662062 | 0.0160203 |
| GPPETE16f004dc | 0.85524938 | 0.02791542 |
| GPPETE16f005dc | 0.7742328 | 0.04881666 |
| GPPETE16f006dc | 0.77531381 | 0.01680798 |
| GPPETE16f007dc | 0.78802609 | 0.01958733 |
| GPPETE16f008dc | 0.81918791 | 0.03502908 |
| GPPETE16f009dc | 0.67202901 | 0.02062262 |
| GPPETE16f010dc | 0.80214524 | 0.00722832 |
| GPPETE18s071R | 0.49609749 | 0.40301029 |
| GPPETE18s072R | 0.66920076 | 0.06832436 |
| GPPETE18s073R | 0.72818831 | 0.11310846 |
| GPPETE18s074R | 0.59861692 | 0.10943131 |
| GPPETE18s075R | 0.533702 | 0.08526994 |
| GPPTCH18s488R | 0.81610591 | 0.14346204 |
| GPPTCH18s489R | 0.81090002 | 0.12440246 |
| GPPTCH18s490R | 0.49317648 | 0.15838425 |
| GPPTCH18s491R | 0.35804081 | 0.2413506 |
| GPPTCH18s492R | 0.33896676 | 0.25701617 |
| GPPTCH18s493R | 0.35916195 | 0.23127053 |
| GPPTCH18s494R | 0.43848545 | 0.16486667 |
| GPPTCH18s495R | 0.41287372 | 0.2196092 |
| GPPTCH18s496R | 0.70075724 | 0.02985413 |
| GPPTCH18s497R | 0.51874145 | 0.12299514 |
| GPPTCH18s498R | 0.47436382 | 0.41445825 |
| GPPTCH18s499R | 0.69179105 | 0.05743681 |
| GPPTCH18s500R | 0.30583699 | 0.29378923 |
| GPPTCH18s501R | 0.57418729 | 0.05941035 |
| GPPTCH18s502R | 0.55627734 | 0.06939831 |
| GPPTCH18s503R | 0.69733287 | 0.05157878 |
| GPPTCH18s504R | 0.80749149 | 0.13617407 |
| GPPTCH18s505R | 0.45922966 | 0.16860364 |
| GPPTCH18s506R | 0.77530969 | 0.09836263 |
| GPPTCH18s507R | 0.65256982 | 0.0989184 |
| GPSALI15f001 | 0.72027815 | 0.07766861 |
| GPSALI15f002 | 0.54181174 | 0.17983597 |
| GPSALI15f003 | 0.67354992 | 0.044462 |
| GPSALI15f004 | 0.68353474 | 0.03403068 |
| GPSALI15f005 | 0.48765839 | 0.02499153 |
| GPSALI15f006 | 0.81272527 | 0.06112022 |
| GPSALI15f007 | 0.50342528 | 0.06906937 |
| GPSALI15f008 | 0.44907481 | 0.11181607 |
| GPSALI15f009 | 0.66503116 | 0.05022732 |
| GPSALI15f010 | 0.69264234 | 0.0409721 |
| GPSALI15f011 | 0.66346352 | 0.10689086 |
| GPSALI15f012 | 0.7570753 | 0.1075137 |
| GPSALI15f013 | 0.49940351 | 0.03867 |
| GPSALI16f001dc | 0.92013291 | 0.00646444 |
| GPSALI16f002dc | 0.89187966 | 0.00511677 |
| GPSALI16f003dc | 0.79872244 | 0.00551673 |
| GPSALI16f004dc | 0.87059577 | 0.00867562 |
| GPSALI16f005dc | 0.89300653 | 0.00360722 |
| GPSALI16f006dc | 0.9199684 | 0.00499026 |
| GPSALI16f007dc | 0.84225515 | 0.09736561 |
| GPSALI16f008dc | 0.90119194 | 0.00320134 |
| GPSALI16f009dc | 0.91467877 | 0.00349113 |
| GPSALI16f010dc | 0.78314456 | 0.0242349 |
| GPSALI17f001 | 0.61458094 | 0.01687397 |
| GPSALI17f002 | 0.43874952 | 0.15715716 |
| GPSALI17f003 | 0.23395624 | 0.05511928 |
| GPSALI17f004 | 0.78890997 | 0.0221317 |
| GPSALI17f005 | 0.79953857 | 0.08047425 |
| GPSALI17f006 | 0.84625042 | 0.03712352 |
| GPSALI17f007 | 0.83552621 | 0.03921296 |
| GPSALI17f008 | 0.80965805 | 0.05611695 |
| GPSALI17f009 | 0.71623713 | 0.03384764 |
| GPSALI17f010 | 0.77267883 | 0.06180453 |
| GPSALI17f011 | 0.76097916 | 0.07910522 |
| GPSALI17f012 | 0.73121713 | 0.12259048 |
| GPSALI17f013 | 0.72625769 | 0.06923153 |
| GPSALI17f014 | 0.27653679 | 0.03884632 |
| GPSALI17f015 | 0.77013055 | 0.05675524 |
| GPSALI18s601R | 0.55592021 | 0.04644545 |
| GPSALI18s602R | 0.82119196 | 0.03914569 |
| GPSALI18s603R | 0.83030654 | 0.02183254 |
| GPSALI18s604R | 0.79583653 | 0.05743455 |
| GPSALI18s605R | 0.75349391 | 0.06833198 |
| GPSALI18s606R | 0.78479862 | 0.07034949 |
| GPSALI18s607R | 0.67650927 | 0.23125092 |
| GPSALI18s608R | 0.84445841 | 0.02935723 |
| GPSALI18s609R | 0.82443825 | 0.01355083 |
| GPSALI18s610R | 0.79009022 | 0.07778937 |
| GPSALI18s611R | 0.80193082 | 0.03945358 |
| GPSALI18s612R | 0.70899661 | 0.06831146 |
| GPSALI18s613R | 0.86803945 | 0.04408301 |
| GPSALI18s614R | 0.8448041 | 0.01572991 |
| GPSALI18s615R | 0.86869194 | 0.04658675 |
| GPSALI18s616R | 0.63554717 | 0.12100534 |
| GPSALI18s617R | 0.81904043 | 0.02891951 |
| GPSALI18s618R | 0.84532795 | 0.0318249 |
| GPSALI18s619R | 0.70223458 | 0.07029802 |
| GPSALI18s620R | 0.84471122 | 0.01973343 |
| GPSALI18s621R | 0.79695444 | 0.04917948 |
| GPSAUN15f001 | 0.66826657 | 0.07872371 |
| GPSAUN15f002 | 0.23475663 | 0.17145947 |
| GPSAUN15f003 | 0.72657701 | 0.02449834 |
| GPSAUN15f004 | 0.44259407 | 0.07359449 |
| GPSAUN15f005 | 0.6055993 | 0.01456393 |
| GPSAUN15f006 | 0.21607582 | 0.0614146 |
| GPSAUN15f007 | 0.66179171 | 0.14611144 |
| GPSAUN15f008 | 0.61685523 | 0.06949894 |
| GPSAUN15f009 | 0.49848381 | 0.00705228 |
| GPSAUN15f010 | 0.32723361 | 0.01958521 |
| GPSAUN16f001dc | 0.68510471 | 0.1039342 |
| GPSAUN16f002dc | 0.7775724 | 0.10026616 |
| GPSAUN16f003dc | 0.73369348 | 0.00788685 |
| GPSAUN16f004dc | 0.8007488 | 0.01408176 |
| GPSAUN16f005dc | 0.88142857 | 0.00354873 |
| GPSAUN16f006dc | 0.77603614 | 0.00273383 |
| GPSAUN16f007dc | 0.65113434 | 0.01446681 |
| GPSAUN16f008dc | 0.71162925 | 0.01746324 |
| GPSAUN16f009dc | 0.75842529 | 0.02089638 |
| GPSAUN16f010dc | 0.77744384 | 0.01469732 |
| GPSAUN18f001R | 0.48261948 | 0.18391685 |
| GPSAUN18f002R | 0.77695647 | 0.08562079 |
| GPSAUN18f003R | 0.69431963 | 0.06529495 |
| GPSAUN18f004R | 0.73188695 | 0.03612927 |
| GPSAUN18f005R | 0.6206838 | 0.00454187 |
| GPSAUN18f006R | 0.75875376 | 0.0193333 |
| GPSAUN18f007R | 0.90722281 | 0.00076609 |
| GPSAUN18f008R | 0.8760268 | 0.01515296 |
| GPSAUN18f009R | 0.81554291 | 0.04495688 |
| GPSAUN18f010R | 0.46687199 | 0.05426706 |
| GPSAUN18f011R | 0.36830189 | 0.05448481 |
| GPSAUN18f012R | 0.75327443 | 0.07161063 |
| GPSAUN18f013R | 0.45202271 | 0.02832715 |
| GPSAUN18f014R | 0.5740097 | 0.06157112 |
| GPSAUN18f015R | 0.58629995 | 0.02773191 |
| GPSAUN18f016R | 0.70525511 | 0.09693851 |
| GPSAUN18f017R | 0.35379962 | 0.07432091 |
| GPSAUN18f018R | 0.85746636 | 0.01406517 |
| GPSAUN18f019R | 0.27495825 | 0.04039998 |
| GPSAUN18sw020 | 0.85192928 | 0.01247754 |
| GPSELV18s020R | 0.54914456 | 0.07650624 |
| GPSELV18s021R | 0.58972391 | 0.03976701 |
| GPSPRT18s024R | 0.37190951 | 0.04424242 |
| WC | 0.73557536 | 0.09423724 |

**^a^** Samples names are ordered by the two or three letter species code, the four letter site ID, and the 2 number year identification, and the individual information.

Supplemental Figure 1. Comparison of Alpha Diversity Measures

Supplemental Figure 1. Plots comparing Observed, Shannon, and Simpson metrics of Alpha diversity as a function of latitude.

Supplemental Figure 2. Histograms of Read Depth Distribution


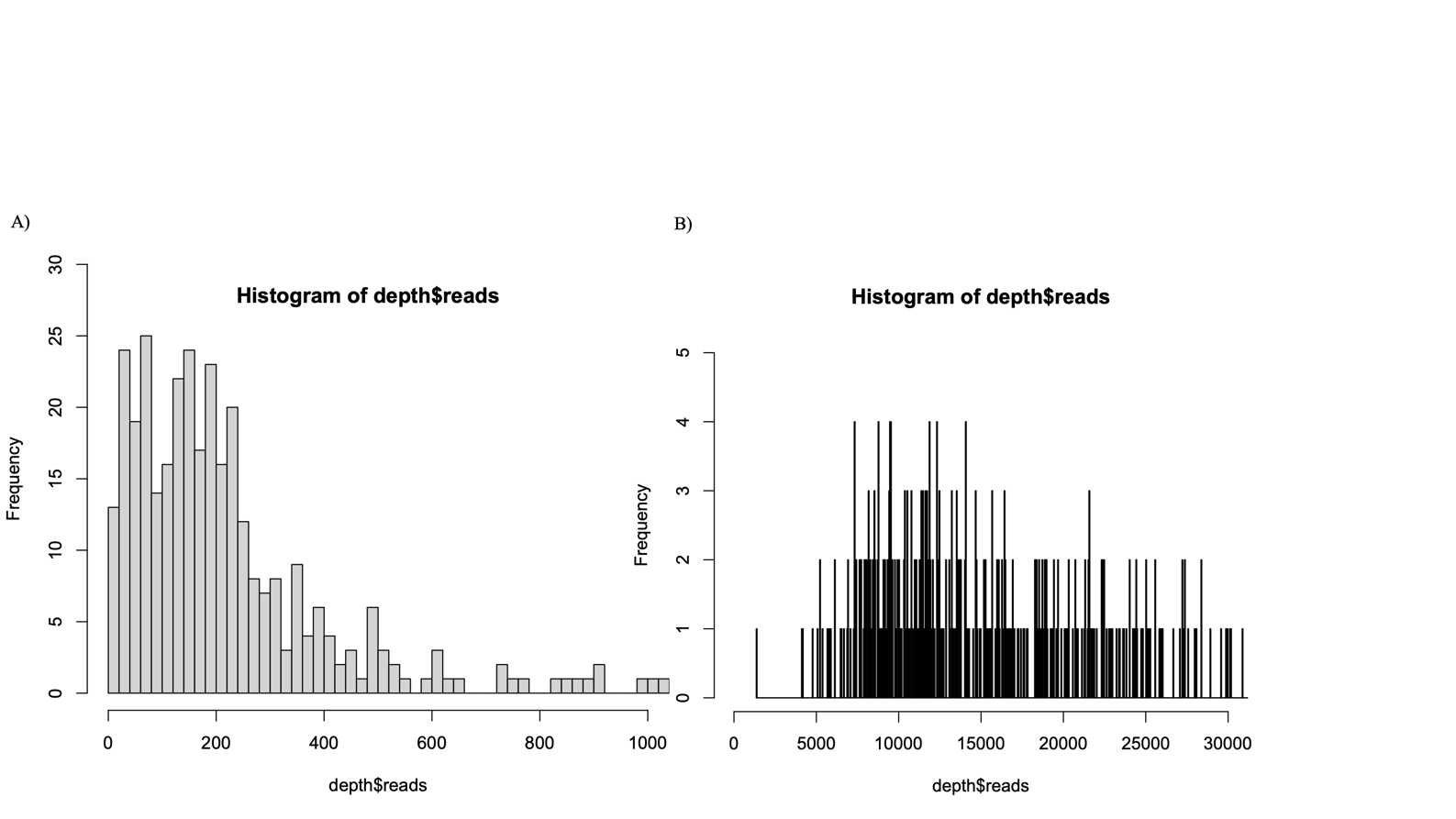


Supplemental Figure 2. Histograms of read depth distribution for A) dietary 18S rRNA gene profiles and B) 16S rRNA gene profiles. For both A and B, the right tail has been cut off.

Supplemental Figure 3. Data Analysis Pipeline

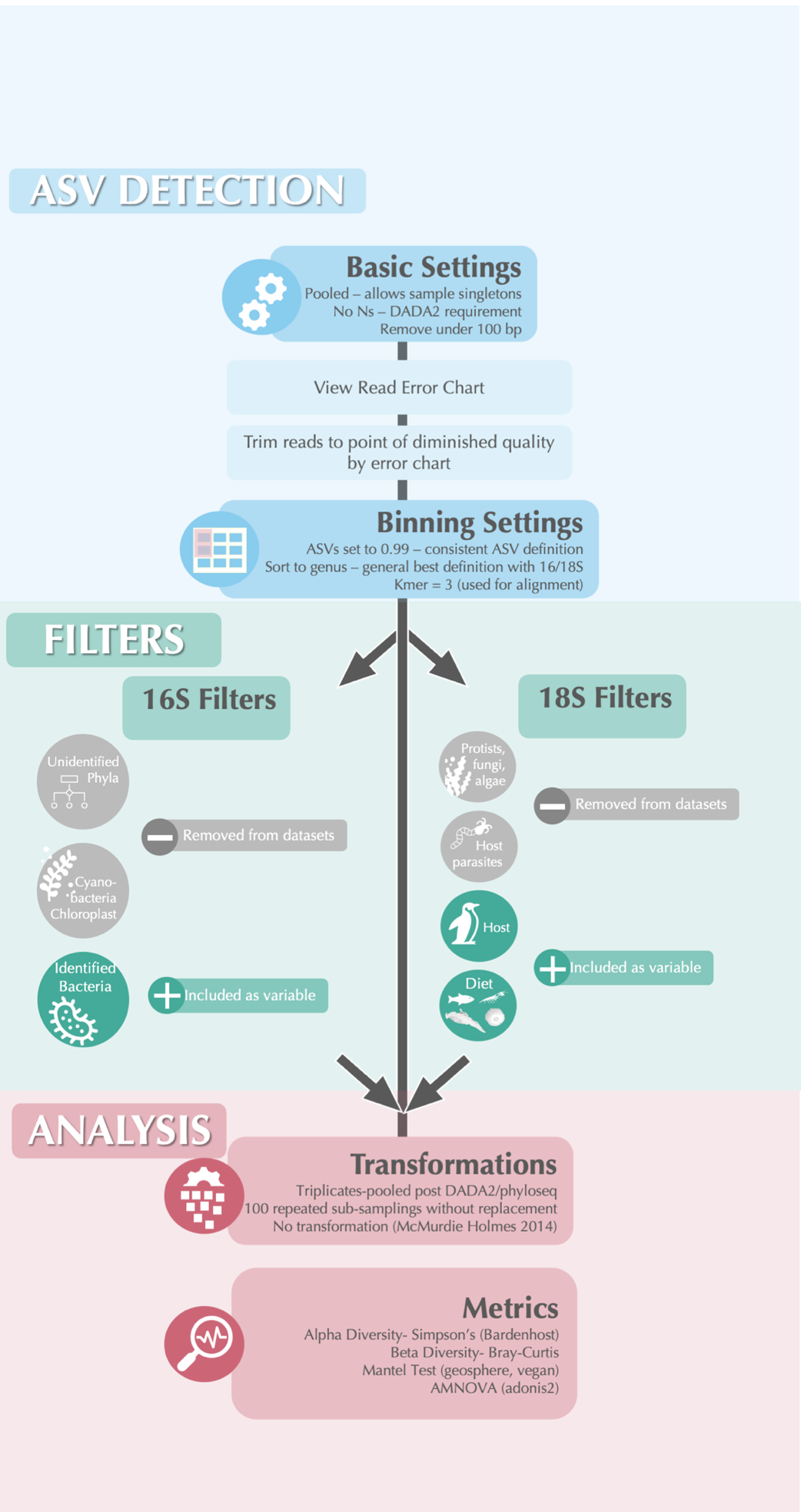


Supplemental Figure 3. A flowchart showing the steps in data analysis, including the various settings used in the processing. For filters, green data (bacteria identified to phylum and the percentage host and all diet information) was kept, while grey data indicates data that was filtered out from the dataset. Metrics also includes the diversity measures and tests used in our analyses.

Supplemental Figure 4. PCoA of 16S rRNA Gene Data


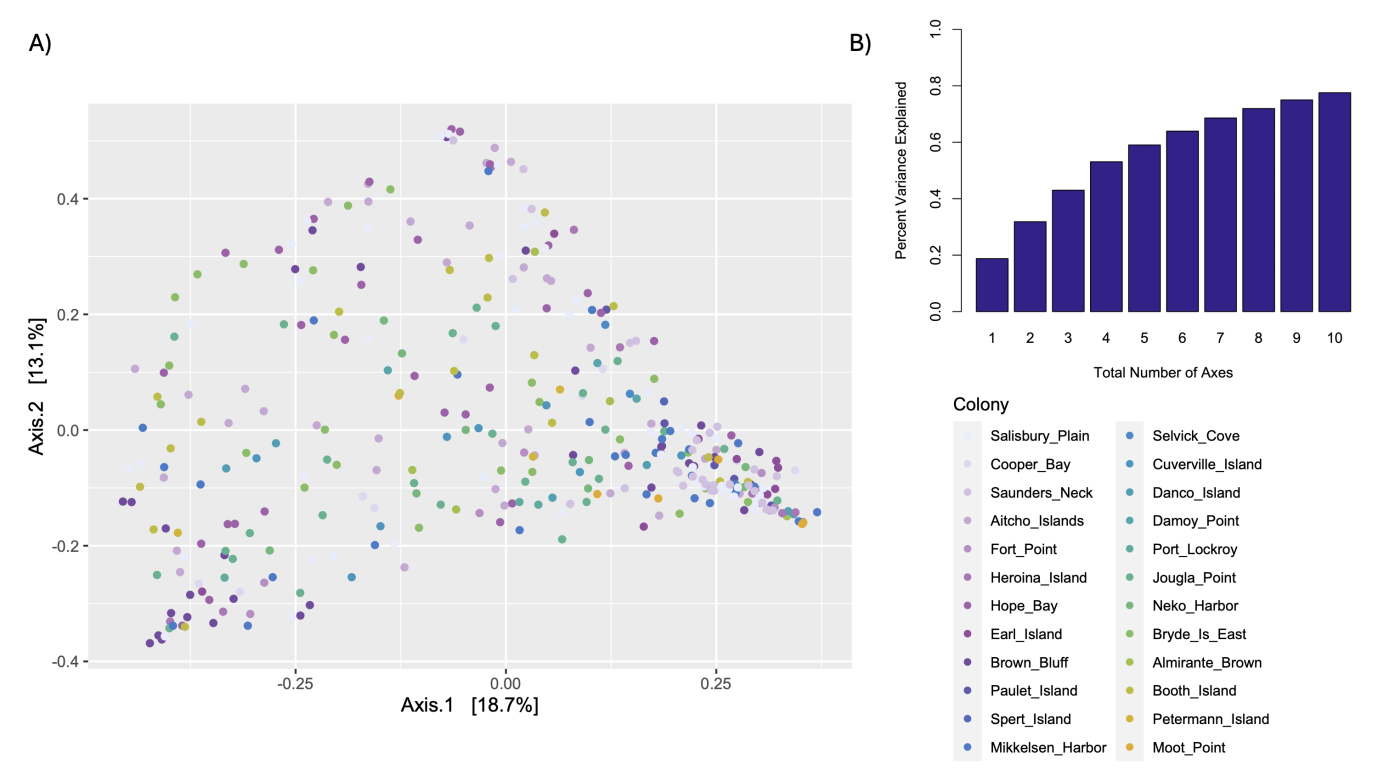


Supplemental Figure 4: PCoA of rarefied 16S rRNA gene data (to 5098 read depth without replacement) using Bray-Curtis, coloured by colony (A). The barplot insert (B) shows the cumulative explained variance for the first 10 axes. The colonies in the reference are arranged in latitudinal order. For other rarefication examples and regional colourings, see Supplemental Figures 5 and 6.

Supplemental Figure 5. Additional PCoAs of 16S rRNA Gene Data


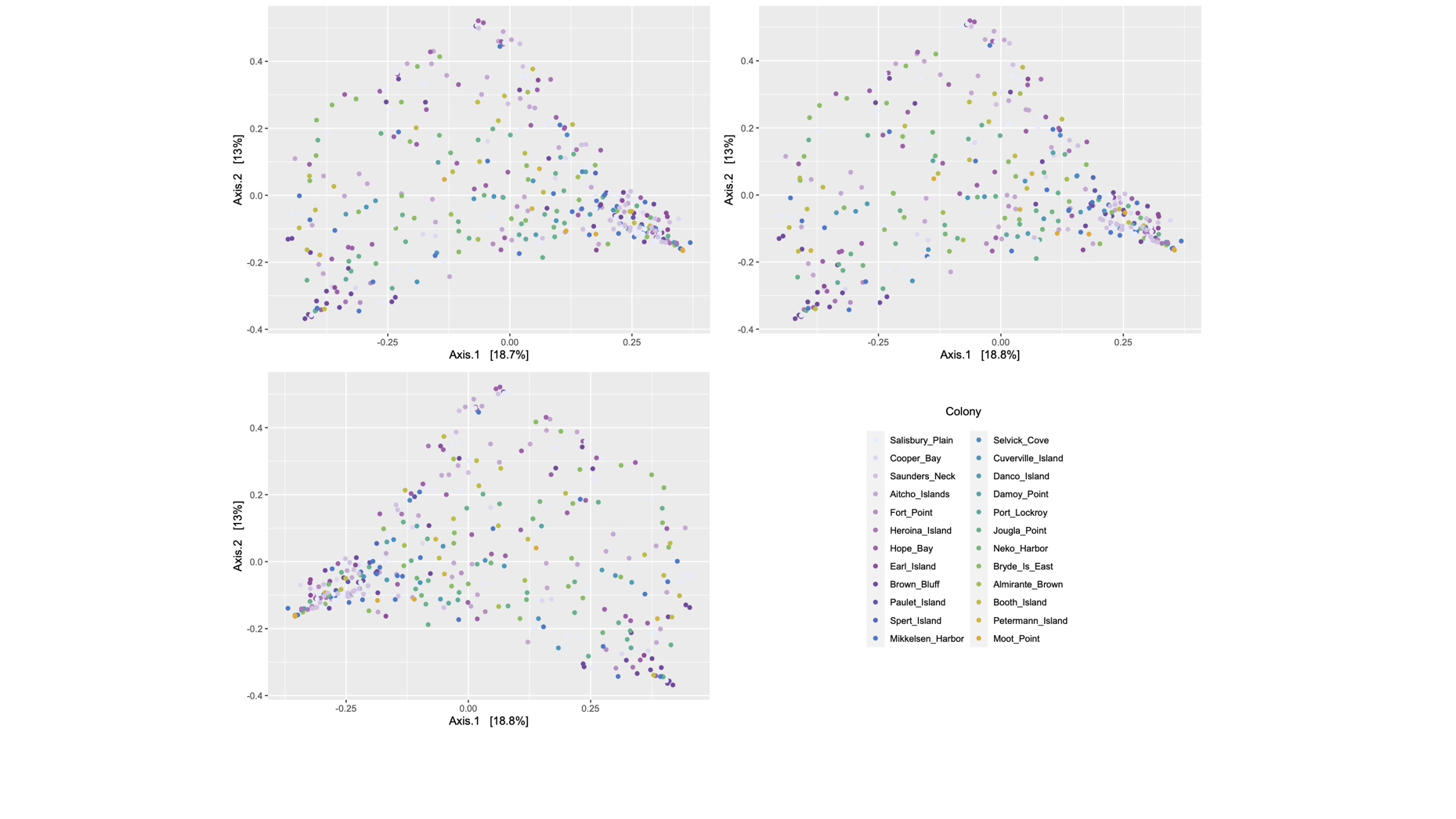


Supplemental Figure 5: 3 additional PCoAs of pseudo-randomly rarefied 16S rRNA gene data (to 5098 read depth without replacement) using Bray-Curtis, coloured by colony. The colonies in the reference are arranged in latitudinal order.

Supplemental Figure 6. Regional PCoAs of 16S rRNA Gene Data


Supplemental Figure 6: PCoAs of pseudo-randomly rarefied 16S rRNA gene data (to 5098 read depth without replacement) using Bray-Curtis, coloured by region.

Supplemental Figure 7. NMDS Visualization of 16S rRNA Gene Data


Supplemental Figure 7: NMDS of 16S rRNA gene data using Bray-Curtis, coloured by colony.

Supplemental Figure 8. NMDS Visualization of 18S rRNA Gene Data


Supplemental Figure 8: NMDS of 18S rRNA gene data using Bray-Curtis, coloured by colony.
